# Downregulated DUXAP8 lncRNA impedes trophoblast cell proliferation and migration by epigenetically upregulating TFPI2 expression

**DOI:** 10.1186/s12958-023-01108-3

**Published:** 2023-06-22

**Authors:** Xiaotong Tang, Yueying Cao, Dan Wu, Lizhou Sun, Yetao Xu

**Affiliations:** 1 Department of Obstetrics and Gynecology, First Affiliated Hospital of Nanjing Medical University, Nanjing, 210029 Jiangsu Province P.R. China; 2 Department of Obstetrics and Gynecology, Women’s Hospital of Nanjing Medical University, Nanjing Maternity and Child Health Care Hospital, 123 Tianfeixiang, Mochou Road, Qinhuai District, Nanjing, 210004 P.R. China

**Keywords:** Preeclampsia, lncRNA, *DUXAP8*, EZH2, TFPI2

## Abstract

**Background:**

Preeclampsia (PE), a pregnancy complication characterized by new-onset hypertension and proteinuria during the second trimester, is the leading cause of neonatal and maternal morbidity and mortality. In the etiology of PE, failure of uterine spiral artery remodeling may be related to functioning abnormally of trophoblast cells, leading to the occurrence and progression of PE. Recently, long noncoding RNAs (lncRNAs) have been reported to play critical roles in PE nowadays. This study aimed to investigate the expression and functions of the TFPI2 pathway-related lncRNA DUXAP8.

**Methods:**

DUXAP8 expression in the placenta from pregnancies was examined using qPCR. Then, the in vitro functions of DUXAP8 were investigated through MTT, EdU, colony, transwell, and flow cytometry experiments. The downstream gene expression profiles were assessed using RNA transcriptome sequencing analysis and verified using qPCR and western blot. Furthermore, Immunoprecipitation (RIP), chromatin immunoprecipitation (CHIP) and fluorescence in situ hybridization (FISH) were used to detect the interaction between lncDUXAP8/EZH2/TFPI2.

**Results:**

The expression of lncRNA DUXAP8 in placenta of patients with eclampsia was significantly decreased. After knockout of DUXAP8, the proliferation and migration of trophoblasts were significantly decreased, and the percentage of apoptosis was increased. Flow cytometry showed that low expression of DUXAP8 increased the accumulation of cells in G2/M phase, while overexpression of DUXAP8 had the opposite effect. We also proved that DUXAP8 epigenetically inhibited TFPI2 expression by recruiting EZH2 and mediating H3K27me3 modification.

**Conclusion:**

Together, these resulting data clarify that aberrant expression of DUXAP8 is involved in the potential PE development and progress. Unraveling the role of DUXAP8 will provide novel insights into the pathogenesis of PE.

**Supplementary Information:**

The online version contains supplementary material available at 10.1186/s12958-023-01108-3.

## Introduction

Preeclampsia (PE) is a syndrome that appears specifically during pregnancy. The main characteristics of PE are edemas, high blood pressure, and proteinuria. The total number of pregnancies affected by PE annually ranges 3%–5% [1]. It is one of the major triggers of fetal morbidity and pregnancy-induced mortality [2]. The disease presents with hypertension and proteinuria, leading to multiple organ dysfunction, including hepatic, renal, and cerebral diseases [3, 4]. Diagnostic laboratory results demonstrate hemolysis, elevated liver enzymes, and decreased platelet count [HELLP (hemolysis, elevated liver enzymes, low platelet count) syndrome] [5]. PE also affects infants as the weight of children born by pregant women with PE is 5% lower than the normal on an average [3]. A family history of PE, hypertension, obesity, chronic kidney disease, and many other maternal comorbidities may lead to PE [6]. Due to limited treatment options, delivery induction is the most effective treatment at present.

The cause of PE remains unclear, and the pathogenesis theory is based primarily on placental dysfunction. Some researchers have proposed the “two-stage” theory. The first stage is the preclinical stage, during which uterine spiral artery trophoblast cell recasting fails. This stage leads to placenta “shallow implantation,” ischemia, hypoxia, and the release of various placental factors. The second stage involves excessive antiangiogenic factors entering the maternal blood circulation, which promotes the activation of systemic inflammation and vascular endothelial damage, resulting in PE, eclampsia, and other various clinical symptoms [3]. Associated animal experimentation has also revealed that uterine placental ischemia leads to hypertension and multiple organ failure [4].

LncRNA is a noncoding RNA with a length of > 200 nucleotides, which lacks a protein-coding function due to the lack of a complete open reading frame [7]. LncRNAs play a crucial role in many disease occurrences and developments, such as cancer [8] and cardiovascular disease [9, 10]. LncRNA expression differs between normal samples and primary tumor samples. The specificity makes it possible for lncRNA to serve as biomarkers or therapeutic targets. The overexpression of the lncRNA MALAT1 has been observed in renal cell carcinoma, gastric cancer, prostate cancer, ovarian cancer, cervical cancer, and lung cancer [11]. The MALAT1 tumorigenesis mechanism involves promoting cell migration [12] and proliferation [13]. LncRNAs are also involved in embryo and organ development and differentiation. The physiological effects of the lncRNA FENDRR are vital for the proper development of the heart and body wall, which are derived from the lateral mesoderm [14]. Mounting studies indicate that lncRNAs affect trophoblast invasion and apoptosis, which influences PE occurrence. Therefore, the identification of PE-associated lncRNAs and related molecular mechanisms is critical for understanding the development of PE and improvement of treatment strategies [15].

LncRNA DUXAP8 is located on chromosome 22q11 and is 2268 bp in length [16]. Several studies have revealed that DUXAP8 participates in the development of various cancers. DUXAP8 is a pseudogene-derived lncRNA that is upregulated in various human cancers and can be used as a biomarker for pan-cancer diagnosis and/or prognosis [17]. It has been reported that DUXAP8 expression is positively associated with the tumor stage and lymph node metastasis [18]. DUXAP8 can also promote the proliferation and migration of gastric cancer cells [18]. In addition, DUXAP8 has been shown to regulate the proliferation and apoptosis of ovarian cancer cells by targeting miR-590-5p [19].

TFPI2 is a matrix-associated Kunitz-type serine protease inhibitor that controls plasmin- and trypsin-mediated activation of zymogen matrix metalloproteinases involved in tumor progression and metastasis, and it was shown that TFPI2 expression inversely correlated with cancer cell invasion and migration [20, 21]. Also, TFPI2 has been reported in several articles on PE. In Wu’article [22], they indicated that linc00473 can inhibit TFPI2 expression by binding to LSD1 in trophoblasts, thus promoting their invasion and migration. Besides, Zheng et al. [23]. TFPI-2 was remarkably elevated in serum and placenta of PE patients, and silencing of TFPI-2 increased the cell invasion and the expression of MMP2 and MMP9, but reduced cell proliferation in HTR-8/SVneo cell line. In summary, TFPI2 was demonstrated to suppress trophoblast cell proliferation and invasion, thus involving in the genesis and progression of PE.

In this study, we found that the RNA levels of DUXAP8 are downregulated in PE placenta tissues. Then we assumed that DUXAP8 may be involved in the pathogenesis of PE. We investigated the trophoblastic phenotype after DUXAP8 knockdown or overexpression. Further associated mechanistic exploration demonstrated that DUXAP8 could exhibit epigenetically regulatory mechanisms in regulation of TFPI2 expression in the nucleus. Unraveling the role of DUXAP8 will provide novel insights into the pathogenesis of PE.

## Materials and methods

### Inclusion population and diagnostic criteria

Pregnant women who undergoing cesarean section at the obstetrics department of the First Affilliated Hospital of Nanjing Medical University were included. Inclusion criteria: single pregnancy, primiparous women without uterine contractions, premature rupture of membranes, or chorioamnionitis clinical diagnosis. Besides, no adverse habits, no history of radiation therapy or chemotherapy. Diagnostic criteria for preeclampsia according to the 2020 AOCG guidelines [24], which includes elevated blood pressure after 20 weeks of pregnancy [systolic blood pressure (SBP) ≥ 140 mmHg or diastolic blood pressure (DBP) ≥ 90 mmHg], proteinuria (24-h urine protein ≥ 300 mg; Protein/creatinine ratio ≥ 0.3: Urinary routine protein (+ +) and above). AOCG guideline also includes patients with hypertension without proteinuria into PE, who were diagnosed as thrombocytopenia, liver and kidney dysfunction, pulmonary edema, etc.

### Tissue collection

Placental tissues were obtained from patients admitted to the First Affiliated Hospital of Nanjing Medical University with an approval. The placental tissue samples were collected from the midsection between the chorionic and maternal basal surfaces at four different placenta locations. We collected eight samples for each placental tissue, and each sample measured approximately 1 cm × 1 cm × 1 cm. The samples were then washed in saline and immediately frozen in liquid nitrogen. Written informed consent was obtained from all patients. This study was approved by the Ethics Committee of the First Affiliated Hospital of Nanjing Medical University.

### Cell culture

All cell lines were provided by Yetao Xu, Department of Obstetrics and Gynecology, First Affiliated Hospital of Nanjing Medical University, China. HTR-8/SVneo and JAR cells were cultured in RPMI 1640 medium (Gibco, Nanjing, China) with 10% fetal bovine serum (FBS) (Invitrogen, Australia), 100 U/mL penicillin, and streptomycin at 37 °C in a humidified atmosphere containing 5% CO_2_.

### Cell transfection

Plasmid vectors (pcDNA-DUXAP8 and empty vector) for transfection were prepared using DNA Midiprep kits (Qiagen, Hilden, Germany). Cell lines were transfected with specific small interfering RNAs (siRNAs), including si-DUXAP8, si-TFPI2, and si-Con. The siRNA sequences are presented in Supplementary Table 1. These cell lines were cultured on six-well plates. The Lipofectamine 3000 transfection Kit (Life technologies, Invitrogen, Carlsbad, CA, USA) was used to transfect cell lines with plasmids and siRNAs according to the manufacturer’s instructions.

### Quantitative real-time PCR

RT-qPCR was performed as previously described by Xu et al. [25]. TRIzol (Invitrogen) was used for cell lysis and RNA precipitation. Using the PrimeScript RT reagent Kit (TaKaRa, Dalian, China), 1 μg RNA was reverse transcribed in a final volume of 20 μL. SYBR Premix Ex Taq (TaKaRa, Dalian, China) was used to determine the expression of DUXAP8 according to the manufacturer’s instructions. All the data were normalized to the expression of GAPDH. After washing and solubilizing the RNA, cDNA was synthesized. PrimeScript RT reagent (TaKaRa, China) was used to obtain the cDNA. SYBR Premix Ex Taq (TaKaRa, China) was used to perform qPCR. All the data were normalized to the expression of GAPDH and ACTB using the comparative 2^−ΔΔCT^ method. The primers sequences used in this study are presented in Supplementary Table 1.

### Subcellular fractionation location

Subcellular fractionation location assays were performed as previously reported by Xu et al. [26]. A PARIS Kit (Life Technologies, USA) was used to separate and purify the cytoplasmic and nuclear RNA according to the manufacturer’s instructions. DUXAP8, U1, and GAPDH expressions in the cytoplasmic and nuclear fractions were determined using qPCR. The results were normalized to the expression of U1 as a nuclear control and to that of GAPDH as a cytoplasmic control.

### Colony formation assay

Colony formation assay was performed to detect cell viability as previously described by Xu et al. [27]. After 48 h of transfection, 600, 800, or 1000 cells were plated and cultured for 10–14 days. To fix the cells, methanol was added, and crystal violet was used to stain them.

### MTT assay

MTT assay was performed as previously described by Xu et al. [27]. Cells were cultured in 96-well plates for about 3 days after transfection. Cells were added with 10 μL MTT and incubated for 4 h. The optical density was measured using enzyme-linked immunosorbent assay.

### EdU assay

EdU assay was performed as previously described by Xu et al. [28]. 5-ethynyl-2′-deoxyuridine (EdU) was used to assess cell line proliferation. The cells were added with EdU solution and incubated for 2 h, following which 0.5% PBS containing TritonX-100 was added to the cells. The cells were stained with Apollo and Hoechst 33342.

### Transwell assay

Migration and invasion assay was performed as previously described by Xu et al. [28]. For the migration assay, 4 × 104 cells were resuspended in 200 µL serum-free medium and added to the upper chambers of transwell plates with 8-μm-pore-sized filter inserts (Corning Costar, Tewksbury, MA, USA); 600 µL complete medium (containing 10% FBS) was added to the lower chambers as a chemoattractant. For the invasion assay, before cells were loaded into the upper chambers, each insert was coated with 1 mg/mL Matrigel (BD Biosciences, San Jose, CA, USA) and incubated at 37 °C for 5 h. Migration and invasion chambers were incubated at 37 °C under 5% CO_2_ conditions for 24 h. After the cells were removed from the culture medium and washed with PBS, they were fixed with 500 µL of 4% paraformaldehyde for 30 min. The inner surface of the filter membrane was wiped using cotton swabs to remove non-migrated or non-invaded cells. The chambers were stained with 500 µL of 0.1% crystal violet for 15 min at room temperature and washed with PBS. Five random microscopic fields of stained cells were observed at 100 × magnification and the cells were counted using ImageJ software (NIH, Bethesda, MD, USA). The invasion experiment is similar to the migration experiment, which was performed as previously described by Xu et al. [28].

### Flow cytometry assay

Flow cytometry assays were performed as previously reported by Xu et al. [26]. After the cells were transfected with siRNAs for 48 h, they were harvested and treated with FITC-Annexin V and propidium iodide (PI) using the FITC-Annexin V Apoptosis Detection Kit (BD Biosciences) according to the manufacturer’s instructions. For cell cycle analysis, cells were stained with propidium oxide using the Cycle TEST PLUS DNA Reagent Kit (BD Biosciences) following the manufacturer’s instructions and evaluated using FACScan. The rate of the cells in each phase were assessed. Cell cycle: the cells were stained with a PI. The cells concentration was detected in G0–G1, S, and G2–M phases, respectively. Apoptosis: the cells were stained with an Annexin V/PI. The apoptotic cells percentage was calculated in the flow cytometry analysis.

### Chromatin immunoprecipitation (ChIP-qPCR)

ChIP assay was performed as previously described by Xu et al. [29]. The cells were transfected and cultured to 85% confluence in a 150-mm dish. Then, 550 μL of 37% formaldehyde was added to 20 ml of growth media to crosslink the DNA for 10 min. A Magna ChIP Kit (Millipore, Billerica, MA, USA) was used for cell lysis. Ultrasonic cell lysis was performed to generate chromatin fragments of 200–300 bp. EZH2, H3K27me3, and IgG were used for TFPI2 immunoprecipitation. Gene expressions were detected by qPCR. The primers sequences are presented in Supplementary Table 1.

### RNA transcriptome sequencing bioinformatics analysis (RNA-seq)

RNA-seq experiments were performed by the Wuhan Genomics Institute (Wuhan, China). RNAs were collected from 3-paired si-NC and si-DUXAP8 transfected HTR-8/SVneo cells, which were isolated using TRIzol reagent (Invitrogen). mRNA extraction was performed using Dynabeads Oligo (dT) (Invitrogen Dynal). Superscript II reverse transcriptase (Invitrogen) and random hexamer primers were used to synthesize complementary double-stranded DNAs. To establish the mRNA-seq library, the cDNAs were fragmented via nebulization by following the standard Illumina protocol.

### RNA immunoprecipitation (RIP–qPCR)

RIP was performed as previously described by Xu et al. [26]**.** RIP experiments were performed using EZ-Magna RIPTM RNA-Binding Protein Immunoprecipitation Kit (Millipore). The lysed HTR-8/SVneo and JAR cells were added to a complete RIP lysis buffer. The cell extracts were immunoprecipitated with a protein A/G magnetic bead conjugated with specific antibodies or control IgG (Millipore) and incubated at 4 °C. The magnetic bead-bound complexes were immobilized with a magnet, and unbound material was washed off with RIP wash buffer. RNAs were extracted according to the manufacturer’s instructions and quantified by qPCR.

### Fluorescence in situ hybridization (FISH)

Trophoblast cells were added with 4% formaldehyde and incubated at room temperature for 10 min. DAPI was used to stain DNA. FISH was performed using a FISH Kit (FISH, RiboBio). The sequence of DUXAP8 RNA-FISH probe was 5′-CAATTCTGGCCTTCTATTCCACAGCAGGGTGACTA-3′.

### Western blotting

Trophoblast cells were cultured for 48 h after transfection. The RIPA cocktail and PMSF was added to prepare a cell protein lysate. The lysate was centrifuged at a high speed (12000 g) for 20 min, and the supernatant was aspirated carefully. A loading buffer was then added to the supernatant and the mixture was heated at 95 °C for 15 min. The running buffer was prepared using tris base, glycine, and SDS. The samples and markers were added to a 12% SDS-PAGE gel (Bio-Rad, Hercules, CA, USA) and run for 90 min. The proteins were then transferred to PVDF membranes (Millipore, USA). The membranes were blocked with skimmed milk for 1 h and incubated with the primary antibody, anti-TFPI2 antibody, and anti-GAPDH antibody (Abcam, Cambridge, MA, USA) at a ratio of 1:1000 overnight. The membranes were washed and incubated with a secondary antibody. The unedited full western blot gels are presented in Supplementary Fig. 1.

### Statistical analysis

GraphPad Prism version 8.0 (San Diego, CA, USA) and SPSS were used to analyze the data. Adobe Photoshop was used to generate images. Data are presented as means ± SD. The data were analyzed using analysis of variance tests or Student’s t-tests (*P < 0.05, **P < 0.01). A *P* value of < 0.05 was used to indicate statistical significance. Each experiment was repeated at least three times independently with similar results.

## Results

### DUXAP8 expression was downregulated in PE

We obtained clinical data of the study participants. Thirty-three paired cases were divided into two groups as follows: 33 PE and 33 normal pregnancies. PE was diagnosed based on new-onset hypertension and proteinuria after 20 weeks of gestation, with a systolic blood pressure (BP) of ≥ 140 mmHg and/or diastolic blood pressure of ≥ 90 mmHg. BP was measured after diagnosis and before treatment. In addition, there were no complications in these cases. Patients with fetal congenital abnormalities, chemical dependency, alcoholism, smoking, diabetes, severe intrauterine growth delay, chronic kidney disease, and primary hypertension were excluded. The clinicopathological characteristics of the participants are summarized in Table 1. In terms of the delivery weeks, PE group was significantly ealier than the control group by nearly 3 weeks (*P* < 0.05), and gestational week had positive correlation with gestational age. In the PE group, accorrding to gestational week with elevated blood pressure, we divided 33 patients into two groups: early onset PE group (*n* = 14) and late onset PE (*n* = 19), the prevalence of small for gestational age (SGA) are 64.3% (9/14) and 36.8% (7/19).

**Table 1:** Clinical characteristics of the patients with and without PE

Variable	PE (*n* = 33)	Control (*n* = 33)	*P* value
Maternal age (years)	31.45 ± 4.53	29.39 ± 3.37	> 0.05
Maternal weight (kg)	75.08 ± 10.1	69.13 ± 9.27	> 0.05
Smoking	0	0	
Systolic blood pressure (mmHg)	153.70 ± 10.88	114.91 ± 10.38	< 0.01^*^
Diastolic blood pressure (mmHg)	99.91 ± 10.85	74.03 ± 8.91	< 0.01^*^
Proteinuria (g/d)	> 0.3	< 0.3	
Body weight of infant (g)	2400.30 ± 664.34	3412.42 ± 504.33	< 0.01^*^
Gestational age (wk)	36.51 ± 2.83	39.47 ± 1.57	< 0.05^*^

^*^P < 0.05

**Fig. 1:**
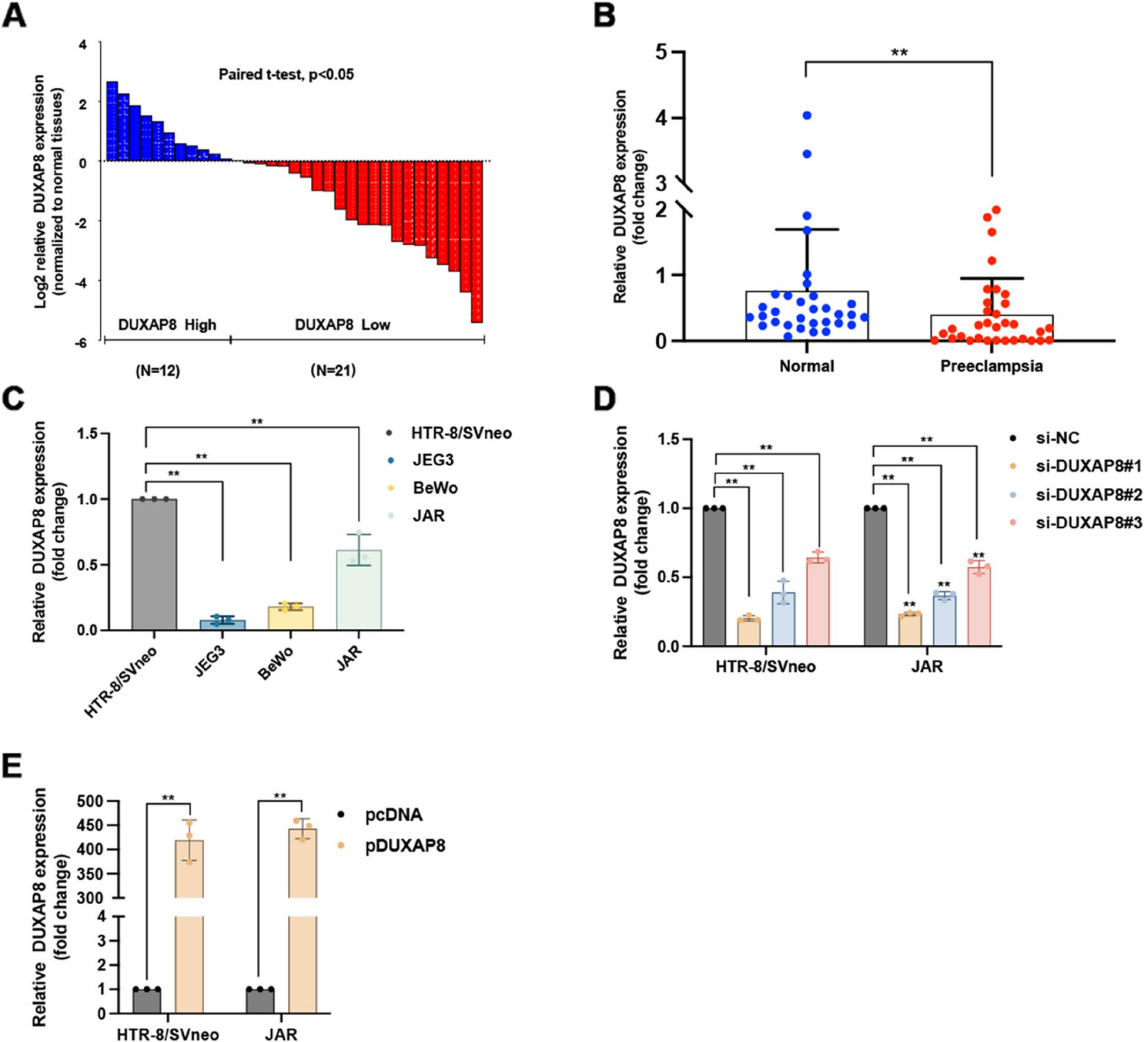
DUXAP8 expression in placental tissues and cell lines. **A** The DUXAP8 expression in placental tissues was tested by the qPCR and was normalized in GAPDH. In each group, the DUXAP8 expression in the PE placenta tissue was compared to the normal control. The data were represented as log2 fold changes (PE/normal, shown as − ΔΔCT) and defined as “ < 0” for underexpression and “ > 0” for overexpression. **B** The DUXAP8 relative expression was presented as the fold change in the PE placental samples compared with the corresponding normal tissues (*n* = 33). **C** The DUXAP8 expression in cell lines was tested by the qPCR. **D** The knockdown DUXAP8 expression was detected by the qPCR after transfecting the JAR and HTR-8/SVneo with a synthesized si-DUXAP8-1#, 2#, 3#. The knockdown DUXAP8 percentage was shown in the figure compared to si-NC. The interference efficiency of #1 reached 85%. **E** The DUXAP8 overexpression was detected by the qPCR after transfecting the JAR and HTR-8/SVneo with a synthesized plasmid pcDNA-DUXAP8 (pDUXAP8). The DUXAP8 relative expression compared to the pcDNA was shown in the Fig. **E**. The multiples of overexpression efficiency reached 400. Data results were expressed as mean ± SEM, *P < 0.05, **P < 0.01

We measured DUXAP8 expression in placental tissues using qPCR. Each PE tissue sample was compared with a normal sample. DUXAP8 expression was significantly decreased in PE tissues compared with the matched normal tissues (Fig. 1A). Moreover, the average expression of DUXAP8 was lower in PE placental tissue than in normal placental tissue (Fig. 1B-C, left panel). Then, the relative DUXAP8 expression in various cell lines (HTR-8/SVneo, JEG3, JAR, and BeWo) was determined using qPCR. JAR and HTR-8/SVneo cells exhibited significantly higher DUXAP8 expression than the other cells (Fig. 1C, right panel).

### DUXAP8 promoted trophoblast cell proliferation, migration and invasion

Three specific siRNAs were synthesized to decrease DUXAP8 expression (Fig. 1D). qPCR analysis was performed 48 h after transfection. The results showed that si-DUXAP8 #2 and #1 were more effective in decreasing DUXAP8 expression than si-DUXAP8 #3. Therefore, si-DUXAP8 #1 and #2 were used for subsequent experiments. Furthermore, DUXAP8 expression increased after transfection with pcDNA-DUXAP8 plasmid (Fig. 1E).

**Fig. 2:**
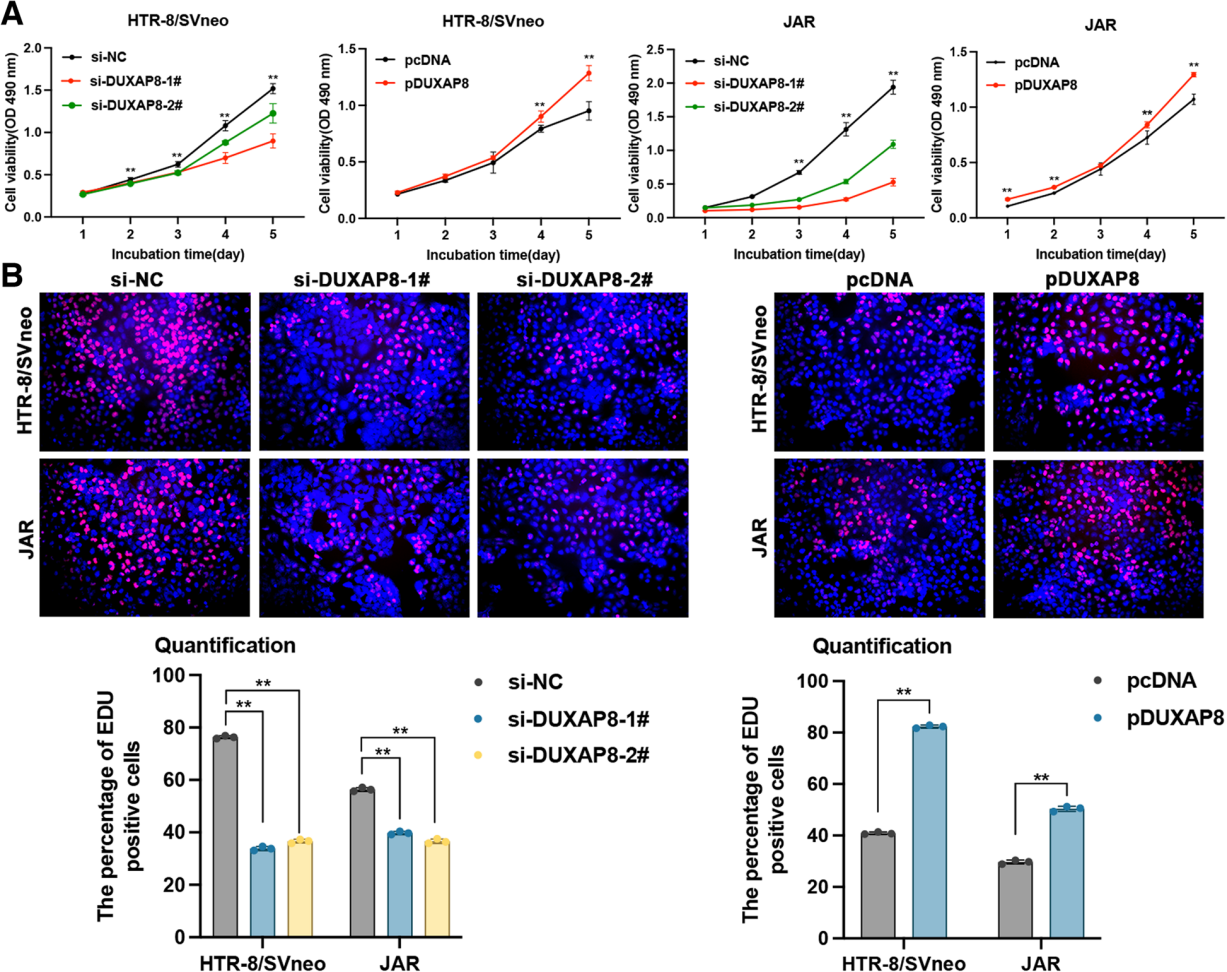
The proliferation of cells were restrained with knockdown DUXAP8. **A** The MTT assays were conducted 5 days in continuous to measure the cells viability after the transfection with si-DUXAP8 and pcDNA3.1-DUXAP8 (pDUXAP8). The knockdown DUXAP8 depressed cell viability and the DUXAP8 overexpression led to the opposite results. **B** The EdU assays were conducted to measure the cells proliferation. The proliferation percentage marked with red was decreased in cells with the knockdown DUXAP8, and the cells dealt with the DUXAP8 overexpression showed the opposite results. Scale bar, 100 μm. Data results were expressed as mean ± SEM, *P < 0.05, **P < 0.01

**Fig. 3:**
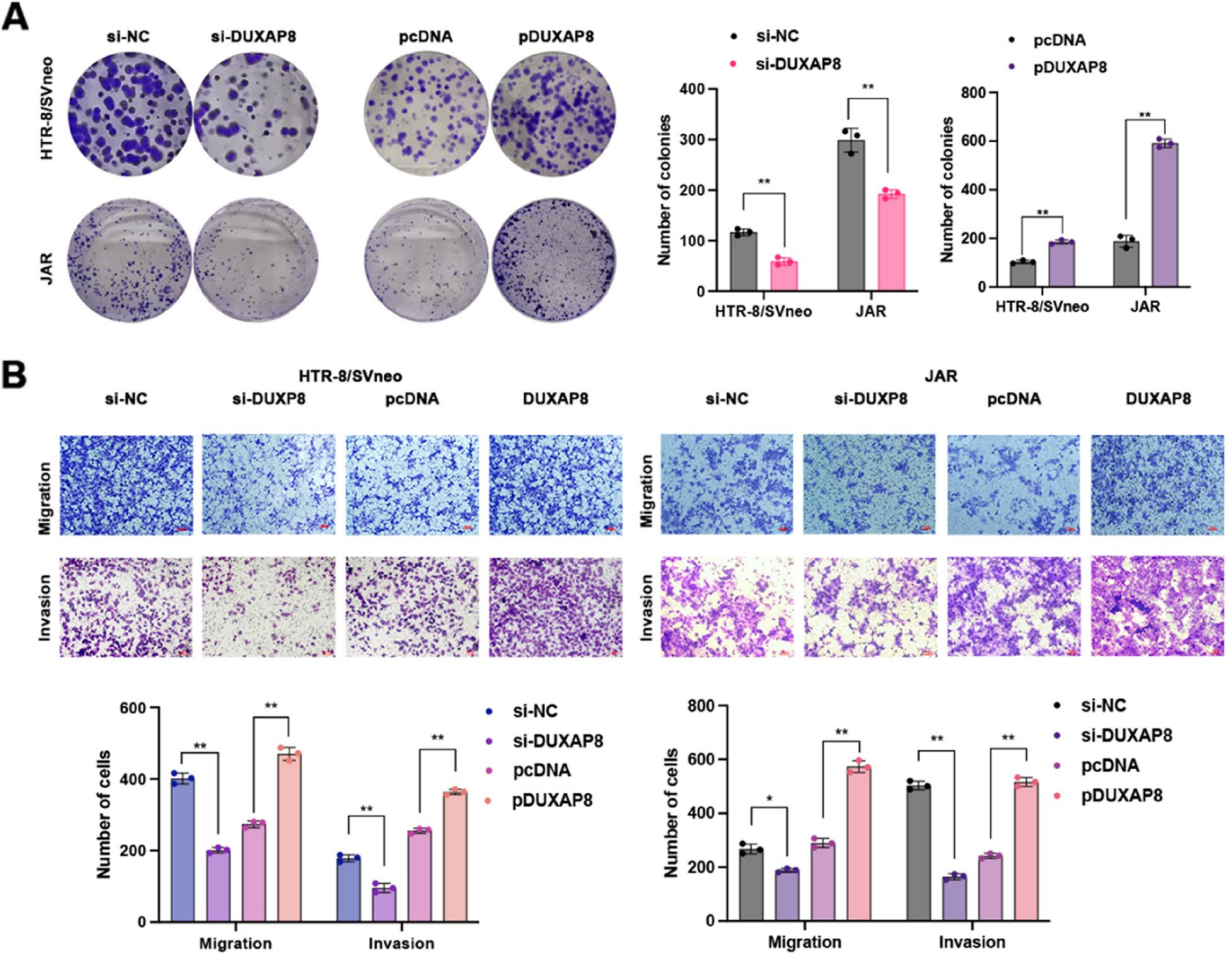
Cell viability, migration and invasion were affected by the DUXAP8. **A** The colony formation assays were conducted to investigate the cells viability after the transfection with the DUXAP8 siRNAs and DUXAP8 plasmid (pDUXAP8). The number of colonies represented the proliferation ability. The viability was depressed in cells with the knockdown DUXAP8. **B** The transwell assays showed that the knockdown DUXAP8 reduced the migrative and invasion ability, and the DUXAP8 overexpression increased the migrative and invasion ability. Data results were expressed as mean ± SEM, *P < 0.05, **P < 0.01

In a previous study, DUXAP8 was reported to promote the proliferation, migration and invasion of ovarian cancer cells [25]. We suspect that the aberrant expression of DUXAP8 plays a role in the occurrence and development of PE by affecting the biological function of trophoblasts. Subsequently, we conducted MTT, EdU, colony formation, and transwell assays to evaluate the effect of DUXAP8 on trophoblast cell proliferation and migration. To investigate the functional regulation of DUXAP8 in trophoblastic cells, we used siRNAs to inhibit endogenous DUXAP8 expression and plasmid to increase DUXAP8 expression. The MTT assay results support the finding that low DUXAP8 expression inhibited HTR-8/SVneo and JAR cell proliferation in a time-dependent manner. DUXAP8 plasmid transfection promoted cell proliferation (Fig. 2A). Through the EdU staining assay, we also validated the decreasing proliferation in correlation with DUXAP8 downregulation, and DUXAP8 upregulation increased spontaneous proliferation (Fig. 2B). As shown in Fig. 3A, cell viability was inhibited by si-DUXAP8 #1 transfection in the colony formation assay. The transwell assays revealed that high DUXAP8 expression was associated with an increased migration and invasion ability in cells, whereas low DUXAP8 expression was associated with decreased migration and invasion ability (Fig. 3B).

### Effects of DUXAP8 on changing the cell cycle and apoptosis of trophoblasts

**Fig. 4:**
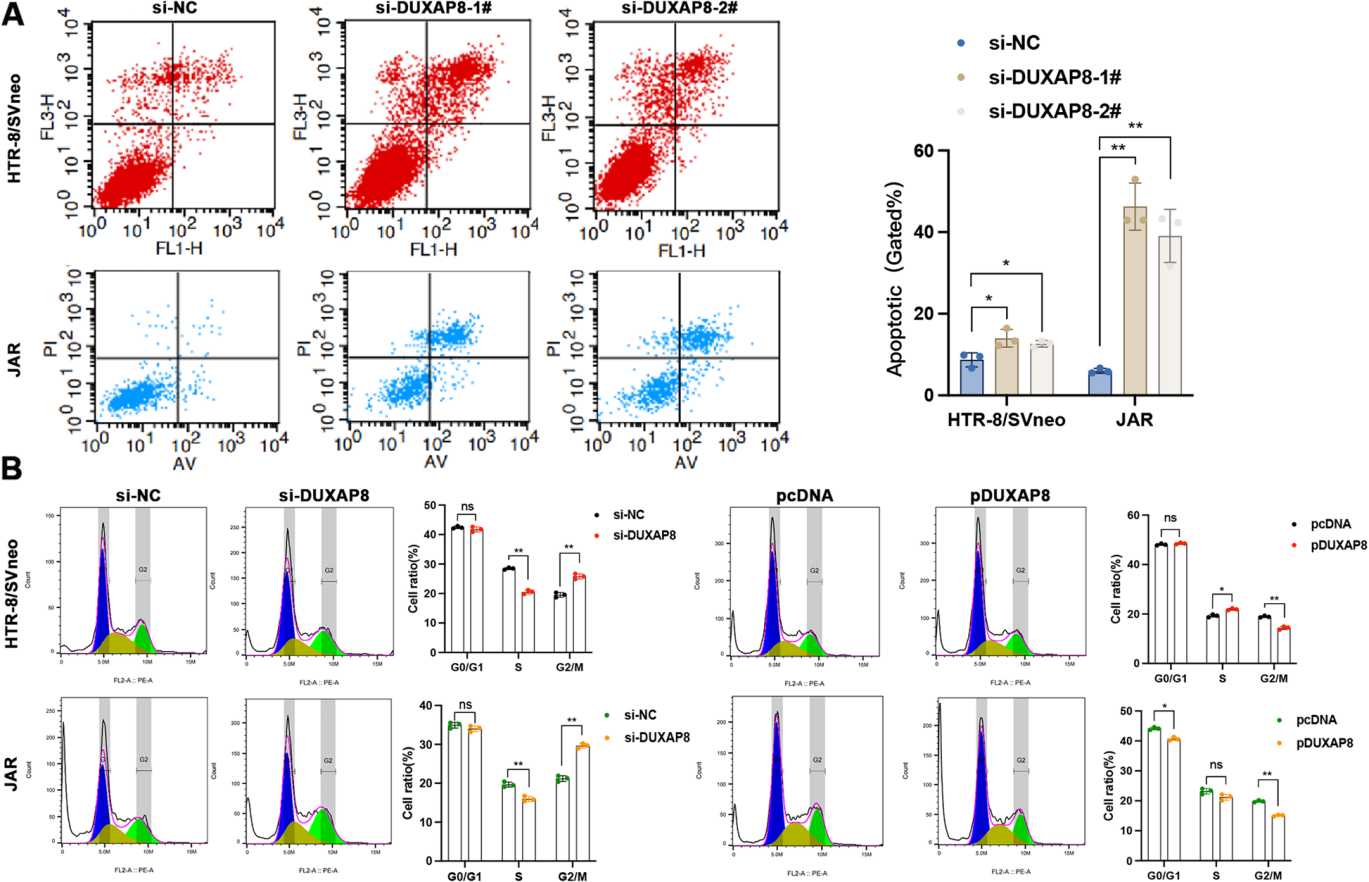
Apoptosis and cell cycles were affected by silencing of DUXAP8. **A** The cell apoptosis was measured by a flow cytometry. The apoptotic cells ratio with the knockdown DUXAP8-1# and DUXAP8-2# was calculated by the LR plus UR after the transfection with the si-DUXAP8 LR, early apoptotic cells and UR, terminal apoptotic cells. **B** A flow cytometry was used to detect the cell cycle. The results showed that different cell concentration appeared in the S and G2/M phrase with the knockdown DUXAP8-1# versus the normal transfection. The G2/M phrase showed differences between the pcDNA and pDUXAP8. Data results were expressed as mean ± SEM, *P < 0.05, **P < 0.01

In order to detect whether the effect of DUXAP8 on cells growth reflects changes in the cell cycle, we conducted flow cytometry to assess the progression of cell cycle. After the cells were transfected by DUXAP8 siRNAs (Fig. 4B). The data revealed that low DUXAP8 expression increased the accumulation of cells in the G2/M phase and decreased it in the S phase, whereas high DUXAP8 expression decreased the accumulation of cells in the G2/M phase. In addition, cell apoptosis was investigated by flow cytometry (Fig. 4A). Annexin V (Ca + -dependent phospholipid binding protein) and PI were used to mark apoptotic cells. The percentage of apoptotic cells marked with Annexin V was remarkably increased in DUXAP8-knockdown cells. These findings suggest that DUXAP8 knockdown alters the cell cycle and induces apoptosis in the cell lines.

### Gene expression profiling

**Fig. 5:**
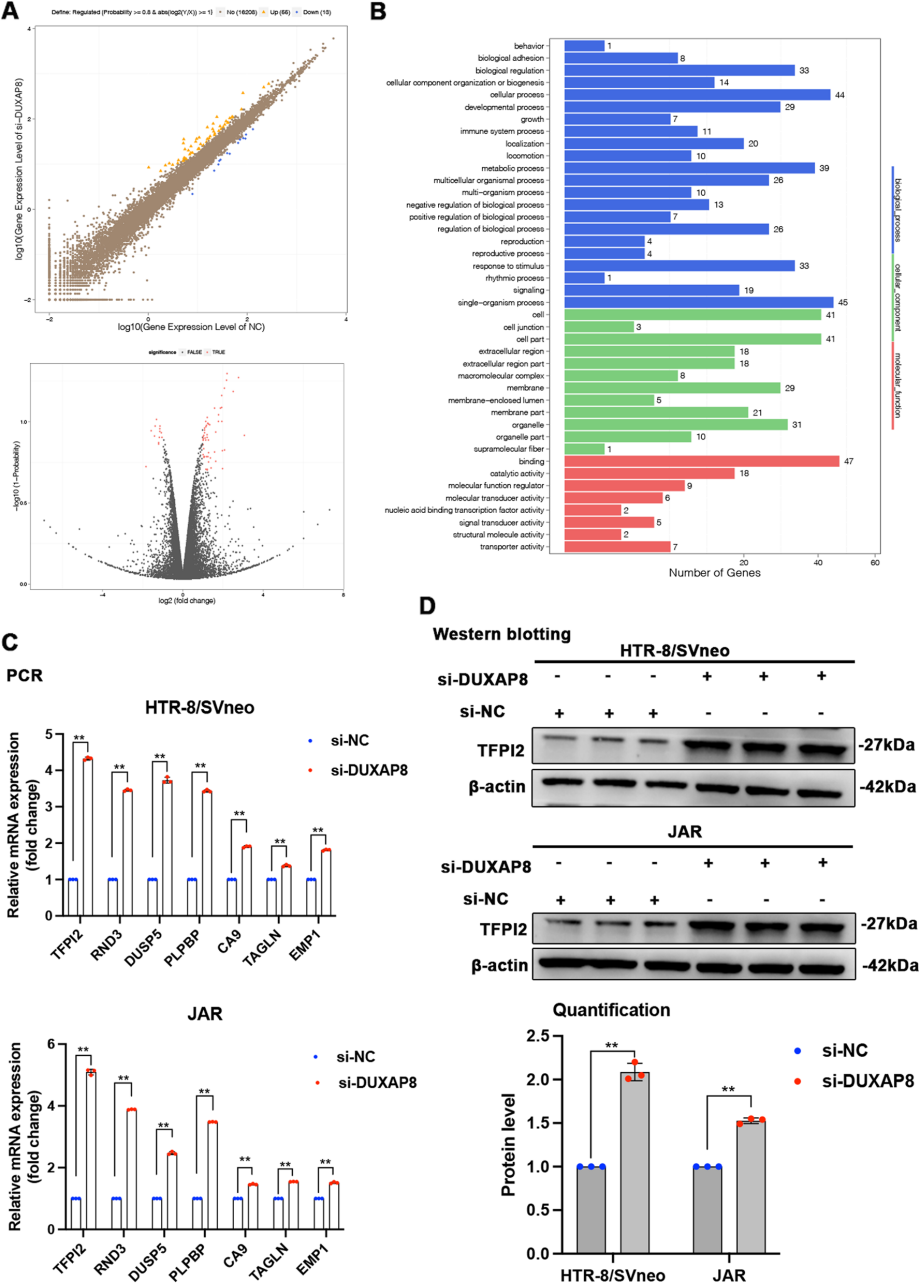
Gene expression profiling. **A** The RNA transcriptome sequencing analysis was performed to analyze gene expression profiling in the HTR-8/SVneo cells following the DUXAP8 knockdown. **B** GO analysis showed different gene expression correlated to the cell growth in cells transfected with the si-DUXAP8. **C** The qPCR assay was conducted to certify the genes related to the DUXAP8. **D** The western blot was conducted to test the protein expression of TFPI2 in the HTR-8/SVneo and JAR cells with the downregulated DUXAP8. The GAPDH was used as a control. Data results were expressed as mean ± SEM, *P < 0.05, **P < 0.01

To test DUXAP8-associated transcriptional changes, we conducted RNA transcriptome sequencing of the control and si-DUXAP8-1#-treated HTR/ SVneo cells to reveal potential downstream targets. The results revealed the latent downstream targets. In HTR/SVneo cells, 55 genes exhibited a ≥ 2-fold increase in transcript abundance after DUXAP8 knockdown, whereas 13 genes exhibited a ≤ 2-fold decrease in transcript abundance (Fig. 5A, Supplementary Table 2). According to Gene Ontology (GO) analysis, the relevant altered genes may have a relationship with proliferation and migration (Fig. 5B). In HTR-8/SVneo and JAR cells, qPCR confirmed the altered genes, including TFPI2, Rho family GTPase 3 (RND3), dual specificity phosphatase 5 (DUSP5), carbonic anhydrase IX (CA9), the epithelial membrane protein genes 1 (EMP1), pyridoxal phosphate-binding protein (PLPBP), and transgenic (TAGLN) (Fig. 5C). Among all the genes, TFPI2 appeared to have the highest expression as observed by qPCR. In addition, we performed a western blot and found the same change in TFPI2 protein expression (Fig. 5D).

### DUXAP8 can affect TFPI2 expression by recruiting EZH2 in the trophoblast’s nucleus

**Fig. 6:**
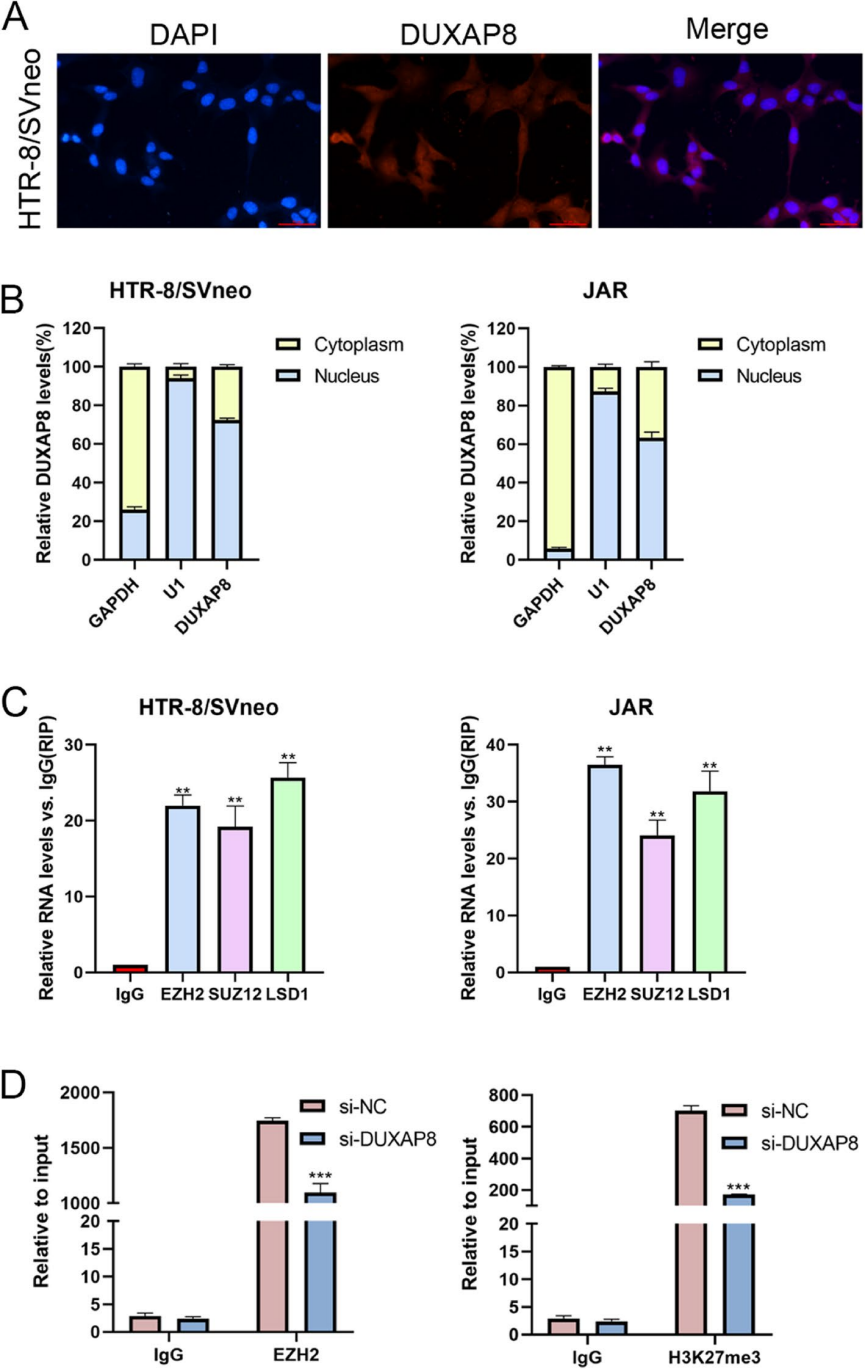
DUXAP8 can affect the TFPI2 expression through recruiting EZH2 in the of trophoblasts nucleus. **A** The FISH assay was also used to detect the DUXAP8 location the in HTR-8/SVneo cells. The results showed that the DUXAP8 was in both the nucleus and the cytoplasm. **B** The cytoplasmic and nuclear RNA isolation assay was conducted to detect the DUXAP8 location in cells. The results showed that the DUXAP8 is in the nucleus of cells mostly. The GAPDH and U1 were used as controls. **C** The RIP was conducted to investigate the possible protein bound to the DUXAP8. The results showed that EZH2, SUZ12, and LSD1 were bound to the DUXAP8. **D** The ChIP was conducted to find the relationship between EZH2, H3K27me3, and TFPI2. The results showed EZH2 and H3K27me3 enrichment in the promoter region of TFPI2. The enrichment was decreased as cells were transfected with the si-DUXAP8. Data results were expressed as mean ± SEM, *P < 0.05, **P < 0.01

To explore the potential biological mechanism of DUXAP8 activity in trophoblast cells, we firstly performed FISH assay to determine the location of DUXAP8 in HTR-8/SVneo cells. The target DNA was labeled with a fluorescent probe based on complementary base pairing. The location of the target DNA can be directly observed using a fluorescence microscope. DUXAP8 was detected both in the cytoplasm and nucleus (Fig. 6A). Then, we conducted a cytoplasmic and nuclear RNA isolation assay to analyze the cellular distribution of DUXAP8. In HTR-8/SVneo cells, approximately 28% of DUXAP8 was localized in the cytoplasm and approximately 72% in the nucleus. In JAR cells, approximately 37% of DUXAP8 was localized in the cytoplasm and approximately 63% in the nucleus (Fig. 6B). We thus speculated that DUXAP8 may be associated with transcriptional regulation. Next, we performed RIP assays to test and verify the proteins bound to DUXAP8. EZH2, suppressor of zest 12 (SUZ12), and lysine-specific demethylase 1 (LSD1) were significantly enriched in DUXAP8 (Fig. 6C). RIP assays were performed both in JAR and HRE-8/SVneo cells.

According to the RIP results, we also assessed the relationship characteristics of the correlation between DUXAP8 and EZH2. Then we performed a ChIP assay, which was used to prepare DNA–protein crosslinks. After the chromatin was cut into small fragments by ultrasound, the antigen and antibody were reacted with the DNA fragments bound to the target protein that was precipitated. As shown in Fig. 6D, the promoter region of *TFPI2* can recruit EZH2 and H3K27me3, and the recruitment was decreased after DUXAP8 knockdown. Therefore, these results indicate that DUXAP8 can stimulate trophoblasts growth and migration partially by inhibiting TFPI2 expression.

### TFPI2 was upregulated in PE and reversed the positive effects of DUXAP8 on trophoblast cells

**Fig. 7:**
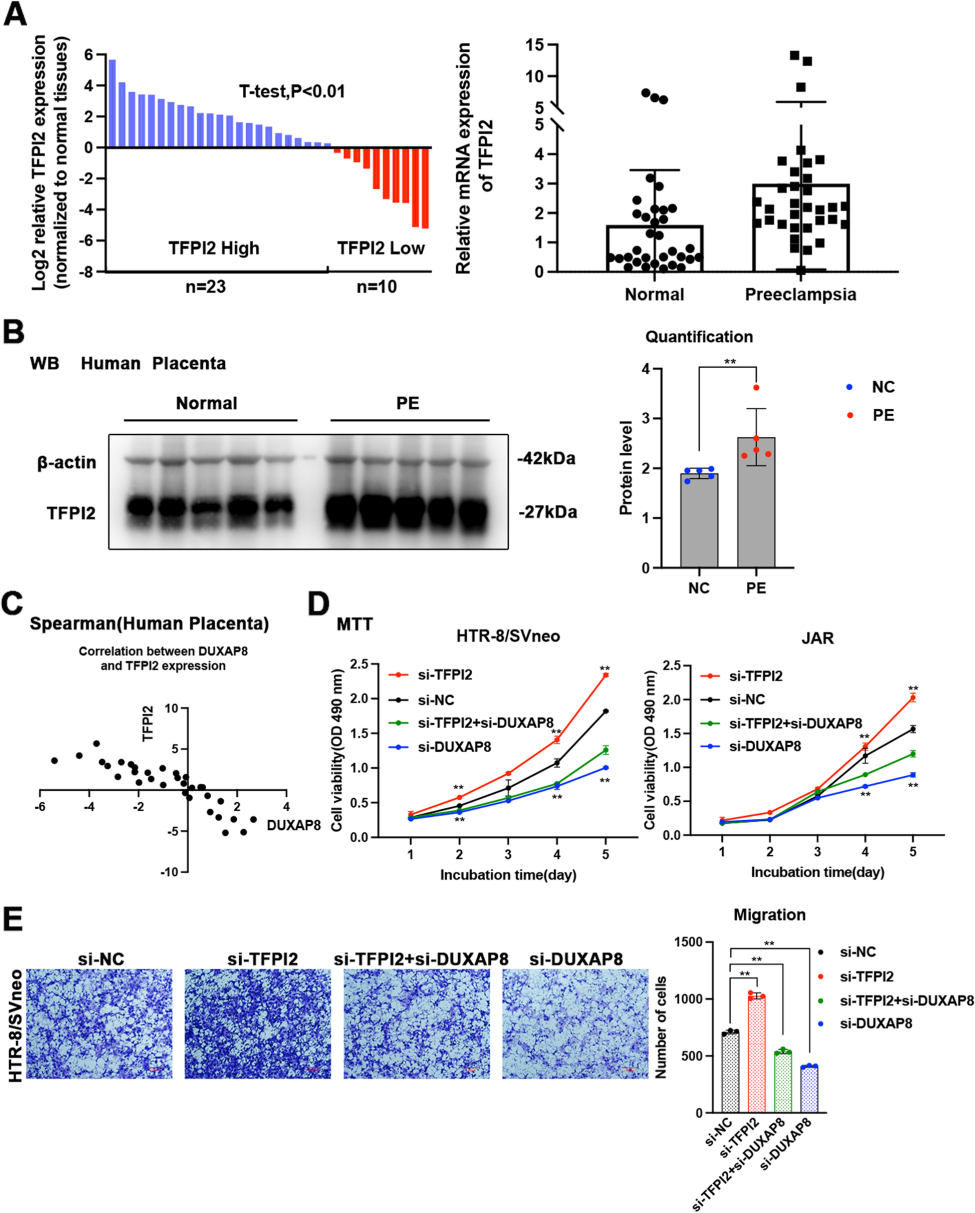
High TFPI2 expression in PE tissues and its effects on trophoblast cells. **A** The TFPI2 expression in placental tissues was tested by the qPCR and normalized to the GAPDH. The results showed that the DUXAP8 expression of 33 PE tissues is lower than the normal tissues. The TFPI2 relative expression in PE placental samples was compared with the normal tissues. **B** The western blot analysis of the expression of TFPI2 in the PE placental and normotensive placental tissues. **C** The Pearson correlation coefficient was conducted to analyze the correlation between the DUXAP8 and TFPI2 expression, *r* = -0.88. **D** The TFPI2 low expression affected cell viability for the si-DUXAP8-transfected trophoblasts. **E** The si-TFPI2 transfection reversed the cells migration transfected with the si-DUXAP8. Data results were expressed as mean ± SEM, *P < 0.05, **P < 0.01

Using qPCR and Western blotting, we found that TFPI2 expression was higher in the PE tissues than in normal tissues as shown in Fig. 7A-B, and a dramatically negative correlation between the levels of DUXAP8 and TFPI2 was observed in human PE placental tissues compared with that in normal placental tissue (Fig. 7C). Furthermore, MTT assays revealed that TFPI2 can inhibit DUXAP8-mediated cell proliferation (Fig. 7D). Transwell assays confirmed that TFPI2 can inhibit DUXAP8-mediated cell migration (Fig. 7E).

In short, our results uncover that DUXAP8 lncRNA can recruit EZH2 protein and epigenetically reduce TFPI2 expression by mediating H3K27 trimethylation in the TFPI2 promoter regions in trophoblasts.

## Discussion

Several studies have reported that lncRNAs, which differ from protein-coding mRNAs, can affect transcription. There are several mechanisms by which lncRNAs can influence chromatin modifications and structure, and thus transcription or other chromatin-related functions. LncRNAs can regulate histone modifications [30], DNA methylation [31], chromatin remodeling [32], transcription factors [33], genome organization [34], and posttranscription [35]. LncRNA inhibits RNA polymerase II in the upstream promoter region and mediates chromatin remodeling and histone modification to affect lncRNA gene expression [36]. LncRNA and protein-coding gene transcripts can form complementary double strands, which prevent splice site recognition and result in the various types of splicing transcription. In addition, lncRNAs form part of a larger RNA–protein complex that alters the protein’s cytoplasmic position [36]. Thus, lncRNAs are crucial to transcriptional and posttranscriptional regulation of gene expression [37], RNA metabolism, translation, transcription, and epigenetic regulation as well as embryonic development, cell autophagy and apoptosis, and cell maintenance and differentiation. Because of its distinct subcellular localization mode, lncRNAs can perform their intended functions [38]. There are many lncRNAs in the nucleus with low primary sequence conservation and low expression levels. This fact further supports the abovementioned findings [39].

Based on existing research, we found that DUXAP8 expression is significantly low in placental tissues of patients with PE. We first explored the function of the lncRNA DUXAP8 in trophoblast cells. Cellular and molecular experiments revealed that DUXAP8 played an important role in trophoblast cell function, including proliferation, migration, cell viability, cell cycle, and apoptosis. Then, gene expression profiles associated with DUXAP8 were evaluated through RNA transcriptome sequencing. In addition, GO enrichment analysis was performed to determine whether the relevant modified genes have a relationship with cell growth. We found that DUXAP8 regulated TFPI2, which is a novel placenta-specific transcript.

TFPI2 has been reported to inhibit migration, invasion, cell viability, and proliferation in many cancer cells [40]. The main TFPI2 mechanism related to the disease is hypermethylation, which has been confirmed in Barrett’s esophagus [41] and gastric cancer [42]. TFPI2 is a metastasis suppressor gene [43] and affects transcription by histone methylation. qPCR and western blot analyses proved that TFPI2 was regulated by DUXAP8 among the related genes. RIP assay revealed three proteins bound to DUXAP8, including EZH2, SUZ12, and LSD1. ChIP assay confirmed that the impaired function of DUXAP8 modified the chromatin state of the TFPI2 promoter. EZH2 and H3K27me3 enrichment were reduced in the TFPI2 promoter after decreasing DUXAP8 expression. DUXAP8 mediates histone modification to affect TFPI2 gene expression. However, many gaps still remain in the current understanding of the function of DUXAP8 and its role in biological mechanisms in PE. Further studies are warranted to elucidate the mechanism of DUXAP8 action that contributes to the pathogenesis of PE.

Several studies have reported that multiple lncRNAs are differentially expressed with advancing gestation in normal pregnancy [44]. In this study, the gestational ages at which placental tissues were collected differed between the PE and control groups, which could have affected DUXAP8 expression. The time-dependent lncRNA changes may change its protein-coding gene targets. More research is needed to rule out the delivery week effect. To summarize, DUXAP8 promotes cell proliferation and migration by epigenetically silencing TFPI2. This study added to our understanding of lncRNAs in trophoblast cells. However, the importance of PE etiology must be emphasized. Furthermore, other underlying DUXAP8 regulatory mechanisms should be investigated further.

## Conclusion

DUXAP8 was confirmed to have an effect on the proliferation, migration, cell cycle, and apoptosis of trophoblastic cells. We found a novel regulatory mechanism involving the DUXAP8–EZH2–TFPI2 axis, which affected the growth of trophoblastic cells. Further studies are needed to elucidate other potential mechanisms by which DUXAP8 participates in the biological functions of trophoblasts in the first or second trimester placenta. Furthermore, DUXAP8 might be a novel molecular target for the diagnosis and treatment of PE in the future.

## Supplementary Information

**Additional file 1: Supplementary Table S1.** The list of PCR/ChIP-PCR primers and siRNAs sequences.

**Additional file 2: Supplementary Table S2.** Analysis of the RNA transcriptome sequencing data.

**Additional file 3: Supplementary Figure S1.** Uncut western gel imagines.

**Additional file 4.**

## Data Availability

The raw data of the study can be obtained from the corresponding author without reservation.

## Abbreviations

PE: Preeclampsia
lncRNA: Long noncoding RNA
EZH2: Enhancer of zester homolog 2
TFPI2: Tissue factor pathway inhibitor 2
RIP: RNA immunoprecipitation
ChIP: Chromatin immunoprecipitation
