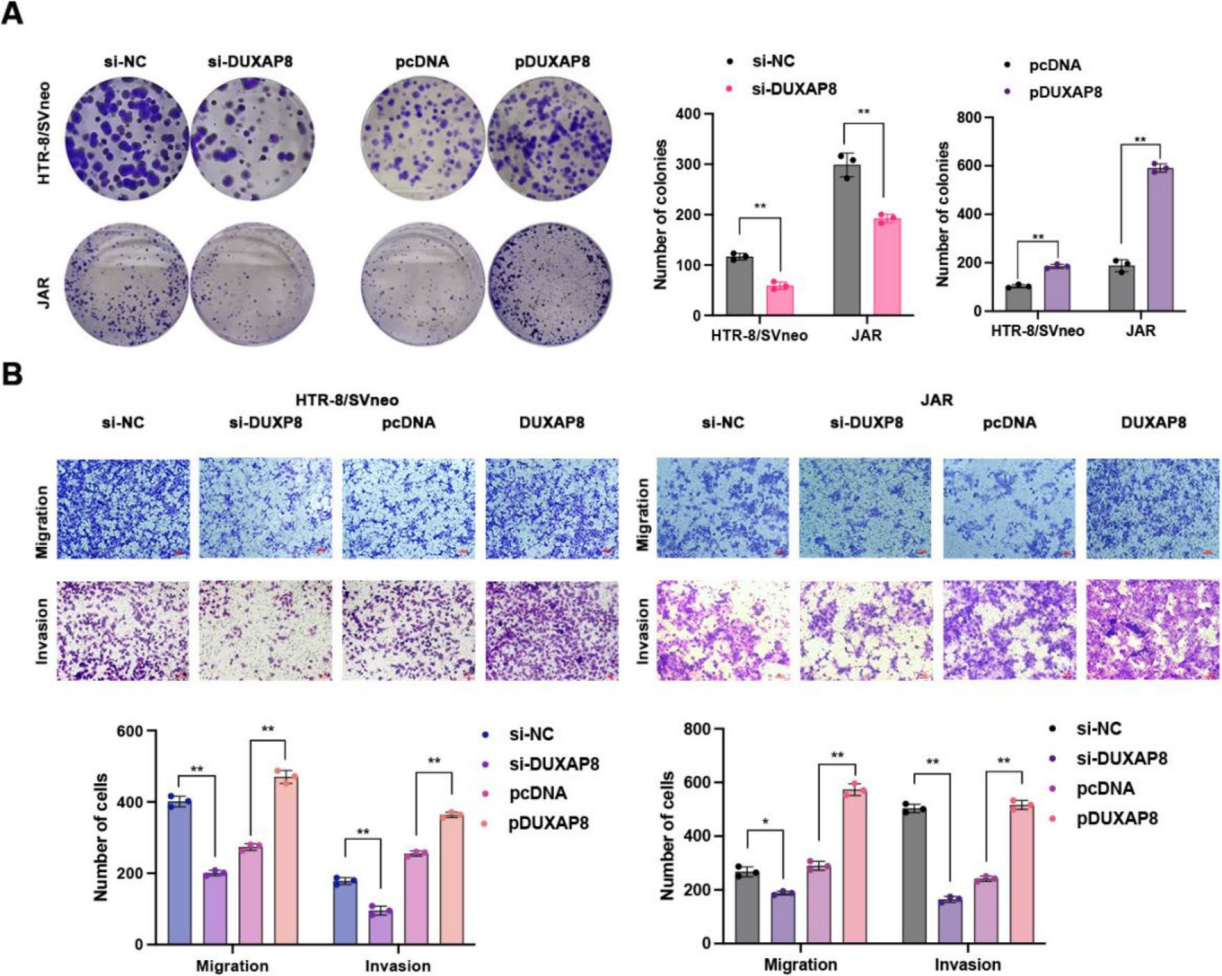# Correction: Downregulated DUXAP8 lncRNA impedes trophoblast cell proliferation and migration by epigenetically upregulating TFPI2 expression

**DOI:** 10.1186/s12958-024-01335-2

**Published:** 2024-12-23

**Authors:** Xiaotong Tang, Yueying Cao, Dan Wu, Lizhou Sun, Yetao Xu

**Affiliations:** 1 Department of Obstetrics and Gynecology, First Affiliated Hospital of Nanjing Medical University, Nanjing, Jiangsu Province 210029 People’s Republic of China; 2 Department of Obstetrics and Gynecology, Women’s Hospital of Nanjing Medical University, Nanjing Maternity and Child Health Care Hospital, 123 Tianfeixiang, Mochou Road, Qinhuai District, Nanjing, 210004 People’s Republic of China

**Correction: Reprod Biol Endocrinol 21, 58 (2023)**

**https://doi.org/10.1186/s12958-023-01108-3**

Following publication of the original article [1], the authors reported an error in the Fig. 3. The Transwell diagram of si NC was mistakenly placed in the position of pDUXP8 Transwell image. The correct Fig. 3 is provided below.

Incorrect Fig. 3


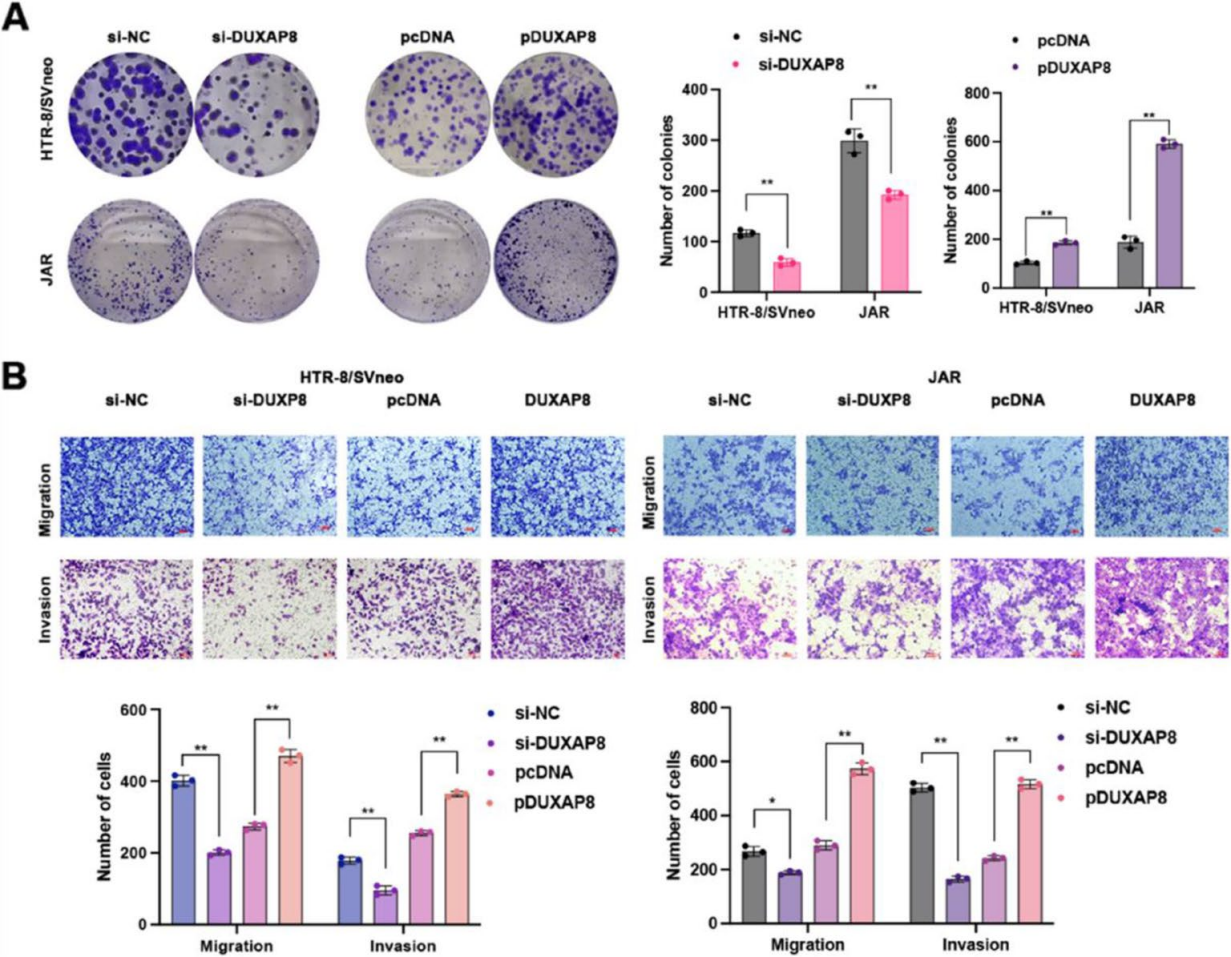


Correct Fig. 3