# Supplementary figures and images for: Downregulated DUXAP8 lncRNA impedes trophoblast cell proliferation and migration by epigenetically upregulating TFPI2 expression
Source: Reprod Biol Endocrinol. 2023 Jun 22;21:58. doi: 10.1186/s12958-023-01108-3 (PMC10286381; doi:10.1186/s12958-023-01108-3)

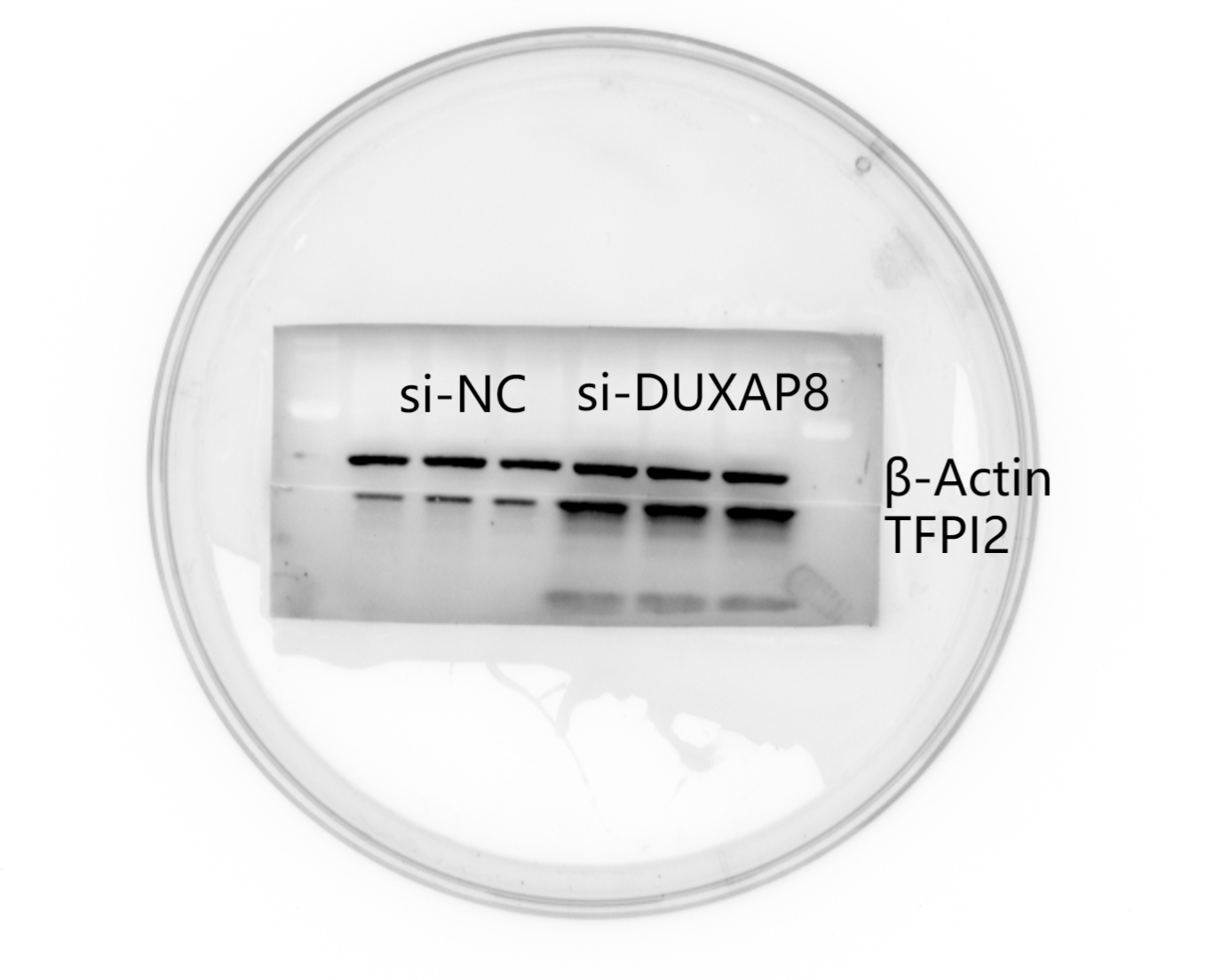

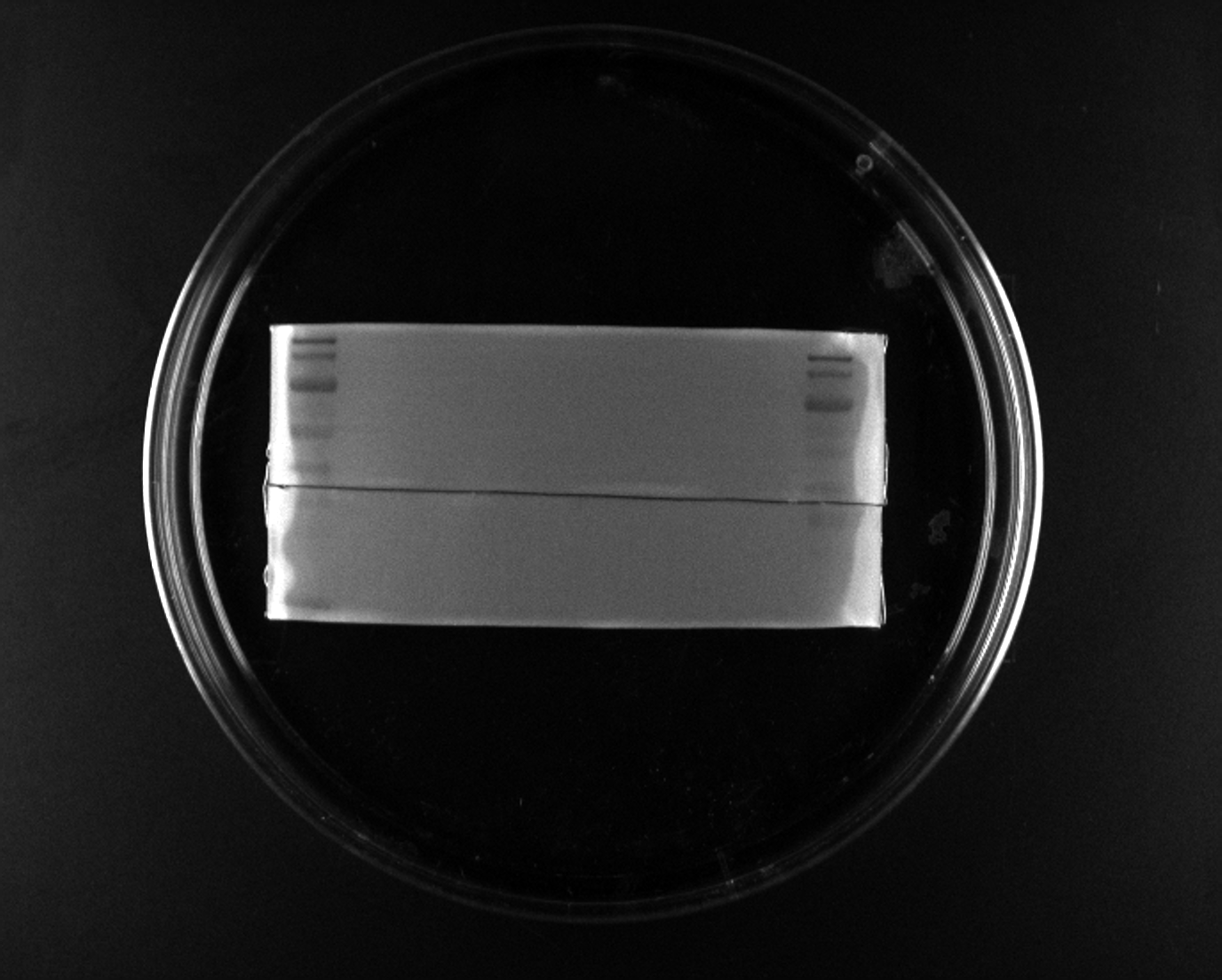


Ladder-1


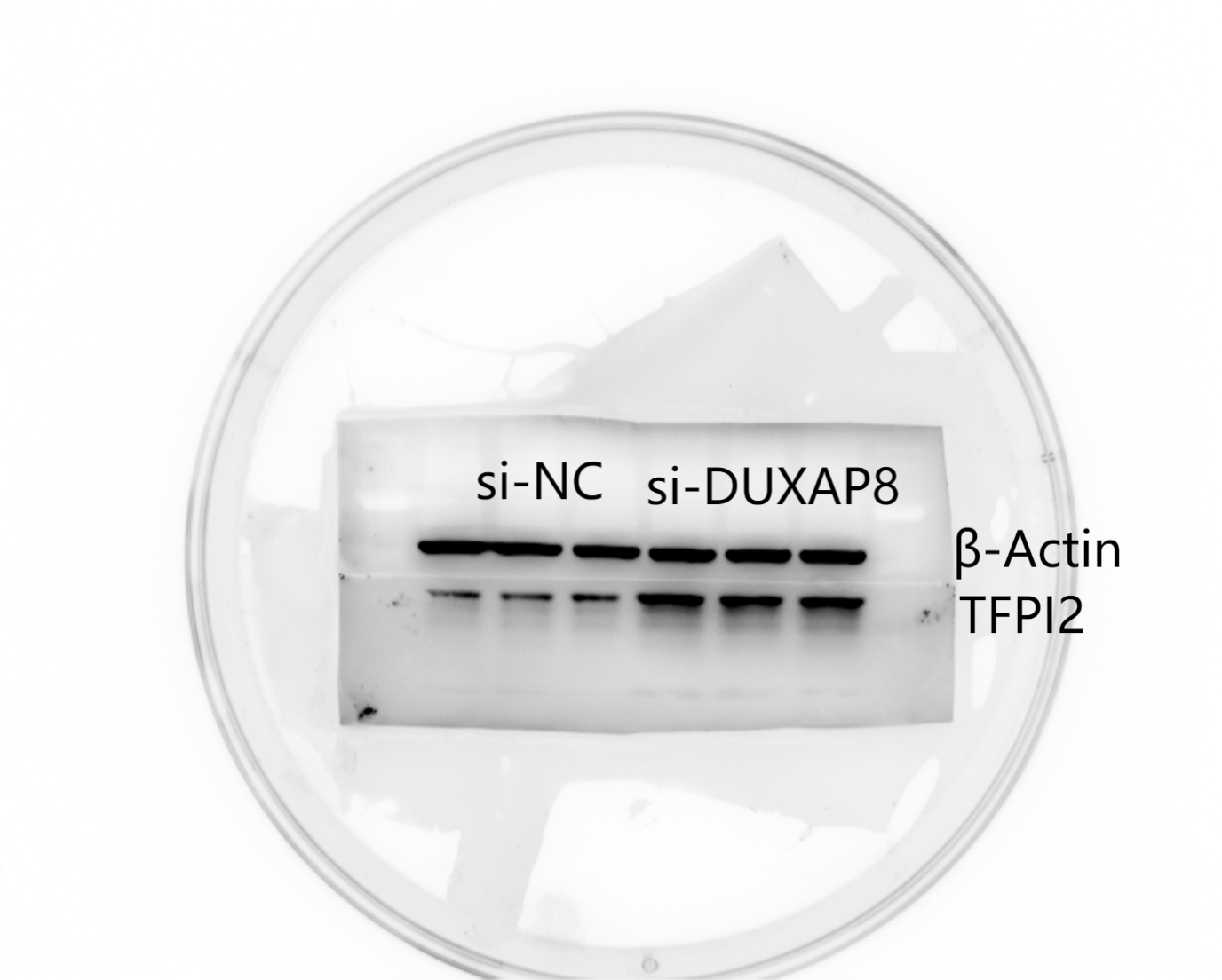

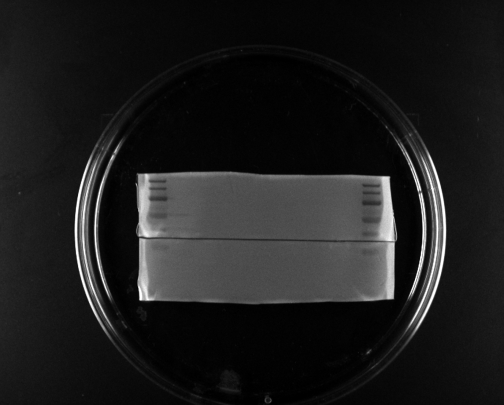

Supplement: Supplementary file 3 — Additional file 3: Supplementary Figure S1. Uncut western gel imagines. [file 12958_2023_1108_MOESM3_ESM.docx]
