# Supplementary material for: Downregulated DUXAP8 lncRNA impedes trophoblast cell proliferation and migration by epigenetically upregulating TFPI2 expression
Source: Reprod Biol Endocrinol. 2023 Jun 22;21:58. doi: 10.1186/s12958-023-01108-3 (PMC10286381; doi:10.1186/s12958-023-01108-3)
Supplement: Supplementary file 4 — Additional file 4. [file 12958_2023_1108_MOESM4_ESM.zip › Additional files.docx]

a.Raw data. Zip

Fig.1

PCR raw materials: DUXAP8 mRNA expression in cell lines and placenta, si-DUXAP8 and pcDUXAP8 CT

Fig.2

MTT materials, and EDU pictures

Fig. 3

Pictures of colon and transwell

Materials of appotosis and cell cycles.

Fig. 5

Name “CT values xlsx”: seveal downstream gene expression in si-nc and si-duxap8 cells

GO, volcano maps of RNA-seq results

Western blot raw pictures.

Fig. 6

qPCR results of CHIP, RIP and related gene primers.

Pictures of FISH

qPCR results of DUXAP8 expression separately in the nucleus and cytoplasm

Fig.7

PCR raw materials: TFPI mRNA expression in placenta, MTT raw materials

Western blot raw pictures

Materials of transwell

Name “pPCR hypoxia and normxia,xlsx”: DUXAP8 and TFPI2 mRNA expression in hypoxia and normxia.

Figure S1: Uncut western gel imagines.
