# Supplementary material for: Downregulated DUXAP8 lncRNA impedes trophoblast cell proliferation and migration by epigenetically upregulating TFPI2 expression
Source: Reprod Biol Endocrinol. 2023 Jun 22;21:58. doi: 10.1186/s12958-023-01108-3 (PMC10286381; doi:10.1186/s12958-023-01108-3)
Supplement: Supplementary file 4 — Additional file 4. [file 12958_2023_1108_MOESM4_ESM.zip › raw data.zip!/raw data/Fig.5/NC-VS-si-DUXAP8.GeneDiffExp.Volcano.pdf]

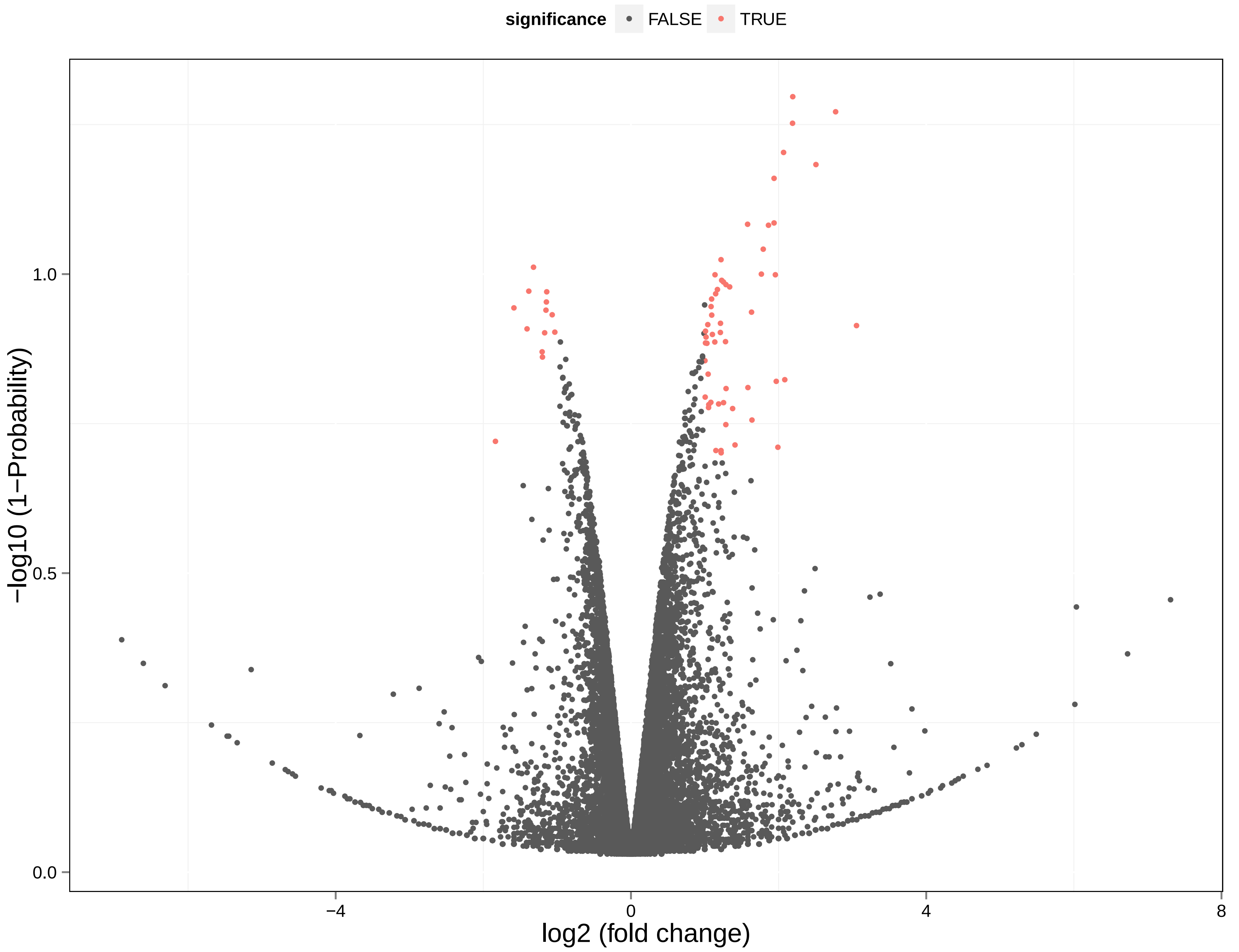
significance
FALSE
TRUE
-log10 (1-Probability)
1.0
0.5
0.0
log2 (fold change)
-4
0
4
8
