# Supplementary material for: Downregulated DUXAP8 lncRNA impedes trophoblast cell proliferation and migration by epigenetically upregulating TFPI2 expression
Source: Reprod Biol Endocrinol. 2023 Jun 22;21:58. doi: 10.1186/s12958-023-01108-3 (PMC10286381; doi:10.1186/s12958-023-01108-3)
Supplement: Supplementary file 4 — Additional file 4. [file 12958_2023_1108_MOESM4_ESM.zip › raw data.zip!/raw data/Fig.5/NoiseqMethod_NC-VS-si-DUXAP8.goclass.pdf]

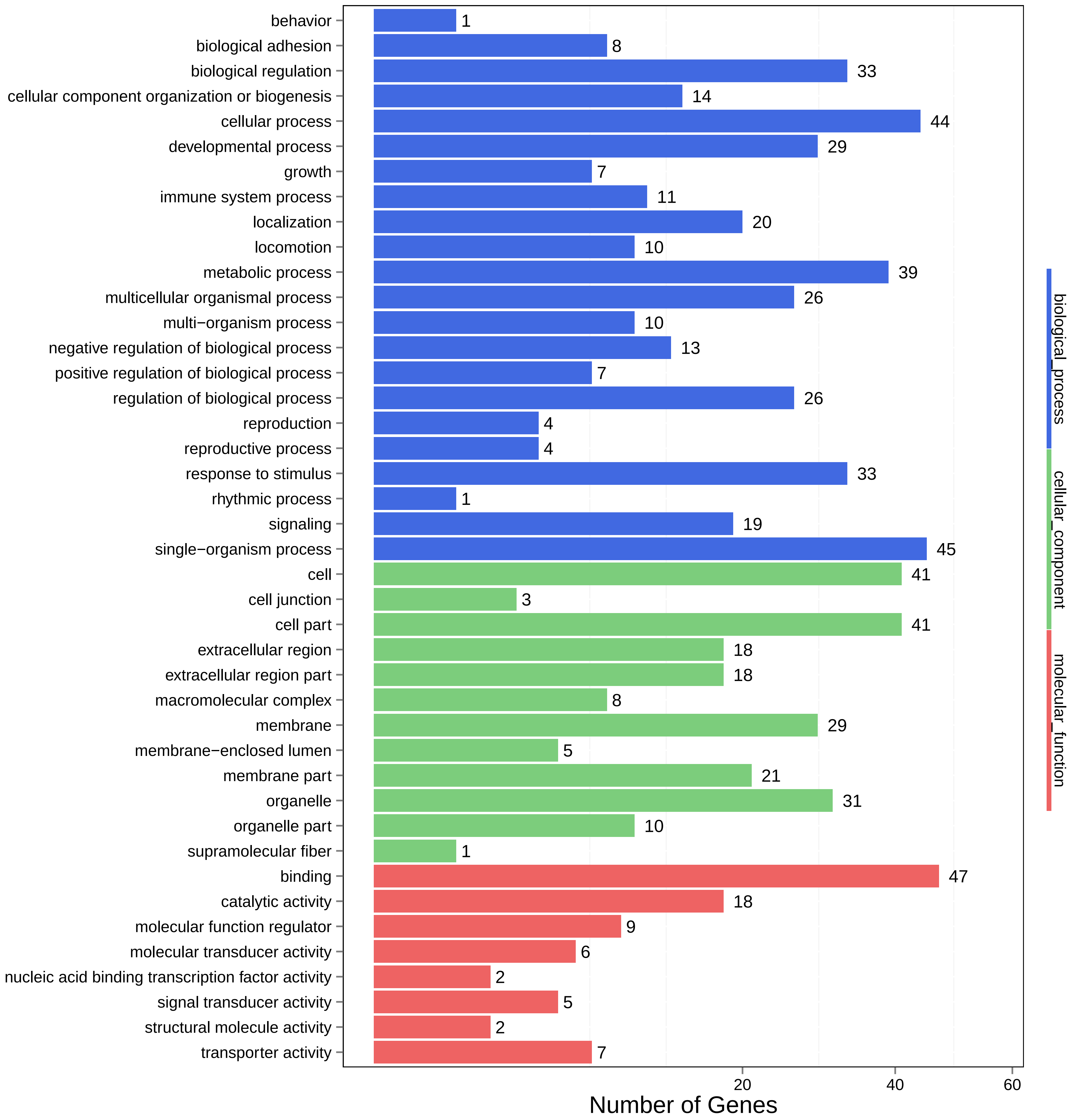

behavior
biological adhesion
biological regulation
cellular component organization or biogenesis
cellular process
developmental process
growth
immune system process
localization
locomotion
metabolic process
multicellular organismal process
multi−organism process
negative regulation of biological process
positive regulation of biological process
regulation of biological process
reproduction
reproductive process
response to stimulus
rhythmic process
signaling
single−organism process
cell
cell junction
cell part
extracellular region
extracellular region part
macromolecular complex
membrane
membrane−enclosed lumen
membrane part
organelle
organelle part
supramolecular fiber
binding
catalytic activity
molecular function regulator
molecular transducer activity
nucleic acid binding transcription factor activity
signal transducer activity
structural molecule activity
transporter activity
1
8
33
14
44
29
7
11
20
10
39
26
10
13
7
26
4
4
33
1
19
45
41
3
41
18
18
8
29
5
21
31
10
1
47
18
9
6
2
5
2
7
20
40
60
Number of Genes
biological_process
cellular_component
molecular_function
