# Supplementary material for: Downregulated DUXAP8 lncRNA impedes trophoblast cell proliferation and migration by epigenetically upregulating TFPI2 expression
Source: Reprod Biol Endocrinol. 2023 Jun 22;21:58. doi: 10.1186/s12958-023-01108-3 (PMC10286381; doi:10.1186/s12958-023-01108-3)
Supplement: Supplementary file 4 — Additional file 4. [file 12958_2023_1108_MOESM4_ESM.zip › raw data.zip!/raw data/Fig.5/NC-VS-si-DUXAP8.GeneDiffExp.pdf]

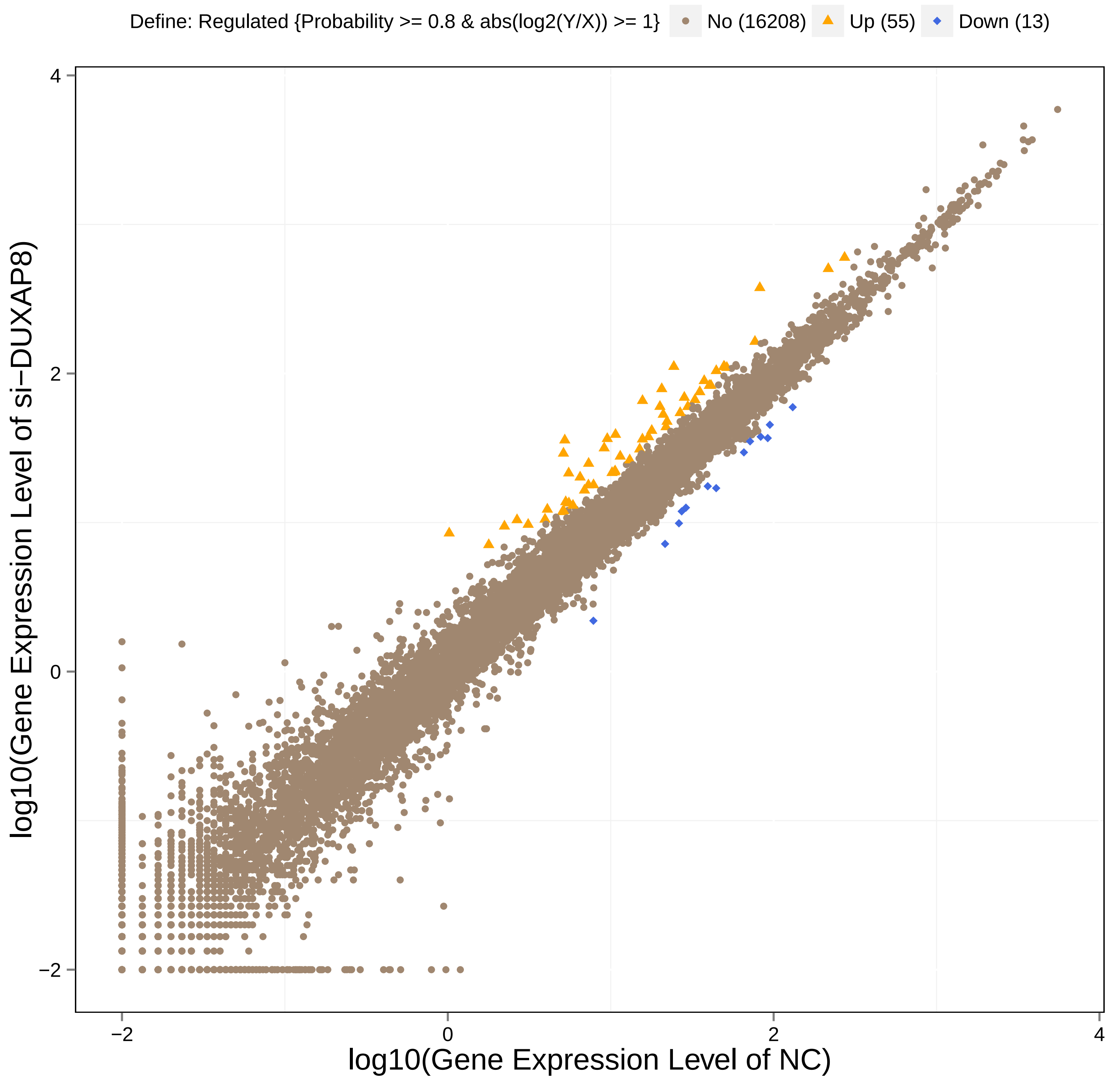

Define: Regulated {Probability >= 0.8 & abs(log2(Y/X)) >= 1}
No (16208)
Up (55)
Down (13)
4
2
0
−2
−2
0
2
4
log10(Gene Expression Level of si−DUXAP8)
log10(Gene Expression Level of NC)
