# Supplementary material for: Downregulated DUXAP8 lncRNA impedes trophoblast cell proliferation and migration by epigenetically upregulating TFPI2 expression
Source: Reprod Biol Endocrinol. 2023 Jun 22;21:58. doi: 10.1186/s12958-023-01108-3 (PMC10286381; doi:10.1186/s12958-023-01108-3)
Supplement: Supplementary file 4 — Additional file 4. [file 12958_2023_1108_MOESM4_ESM.zip › raw data.zip!/raw data/Fig.6/primers.doc]

si-DUXAP8#1：TGTGGATGAACAGGCTAGT；

si-DUXAP8#2： TGAACAGGCTAGTCACCCT；

si-DUXAP8#3：AGGATGGAGTCTCGCTGTA

si-TFPI2：UCUACUGGCAAAGCGAAGCUUUGGC

Primers:

GAPDH (F: GAAGGTGAAGGTCGGAGTC, R: GAAGATGGTGATGGGATTTC);

ACTB (F: GTCCACCTTCCAGCAGCAGATGT, R: CACCTTCACCGTTCCAGTTT);

DUXAP8 (F: TACCATGCTGTCCTCCAGGT, R: GAGCTTGCAGTGAGCTGAGA);

TFPI2 (F: GATGTCGATTCTGCTGCTTT, R: CAAGCCTCCCAGGTGTAGAA);

RND3 (F: GCCAGCCAGAAATTATCCAG, R: AGTGTCCCACAGGCTCAACT);

DUSP5 (F: AGCAACGTGGGAGAAAGAAG, R: CAGGGTTCAAATCAGGATGC);

PLPBP(F: GCAATGGCTTTGGATTGAGT, R: AATCCCTCTGACTTCCACGA);

CA9 (F: GTCTCGCTTGGAAGAAATCG, R: AGAGGGTGTGGAGCTGCTTA);

TAGLN (F: GGGTTCATTCATGGTTGGTT, R: AGAGGGTGTGGAGCTGCTTA);

EMP1 (F: CACCTCAGTGTGGACTTAATCG, R: TTGGAACTGGGTAACGGGTA)
