# Supplementary material for: Downregulated DUXAP8 lncRNA impedes trophoblast cell proliferation and migration by epigenetically upregulating TFPI2 expression
Source: Reprod Biol Endocrinol. 2023 Jun 22;21:58. doi: 10.1186/s12958-023-01108-3 (PMC10286381; doi:10.1186/s12958-023-01108-3)
Supplement: Supplementary file 4 — Additional file 4. [file 12958_2023_1108_MOESM4_ESM.zip › raw data.zip!/raw data/Fig.4/cell cycles/30-Dec-2020-Layout-Batch.pdf]

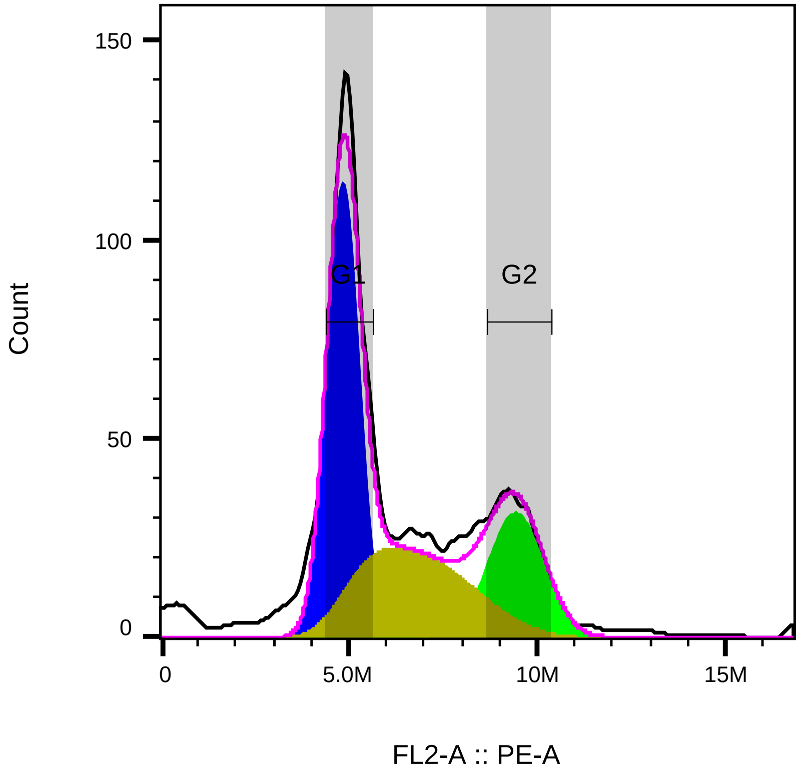

1.fcs
Cell Cycle
5096

RMSD : 1.28
%G1 : 42.1
%S : 28.5
%G2 : 19.6
G1 Mean : 4.86E6
G2 Mean : 9.43E6
G1 CV : 12.6
G2 CV : 11.0
% less G1 : 5.39
% greater G2 : 1.28

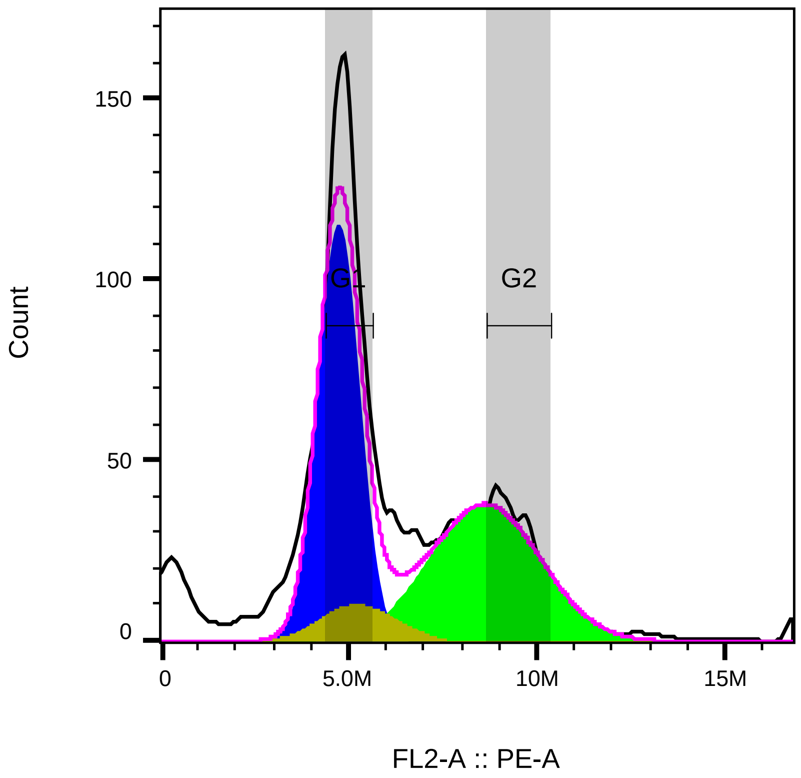

2.fcs
Cell Cycle
6622

RMSD : 2.33
%G1 : 43.7
%S : 5.87
%G2 : 38.8
G1 Mean : 4.76E6
G2 Mean : 8.62E6
G1 CV : 16.1
G2 CV : 23.9
% less G1 : 8.15
% greater G2 : -0.28

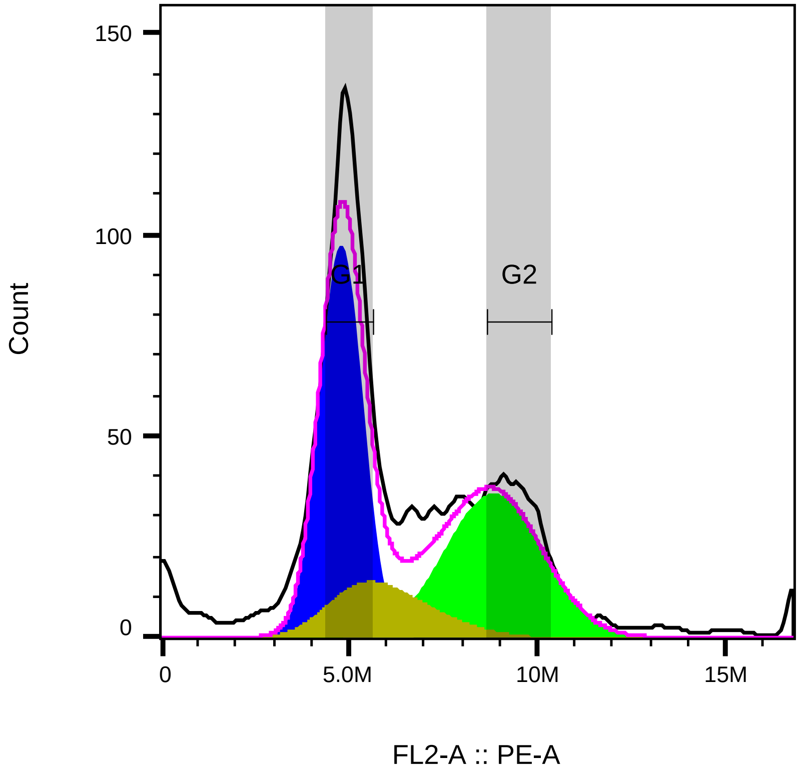

3.fcs
Cell Cycle
6153

RMSD : 1.81
%G1 : 41.5
%S : 13.4
%G2 : 34.9
G1 Mean : 4.81E6
G2 Mean : 8.85E6
G1 CV : 16.5
G2 CV : 20.7
% less G1 : 4.76
% greater G2 : 2.36

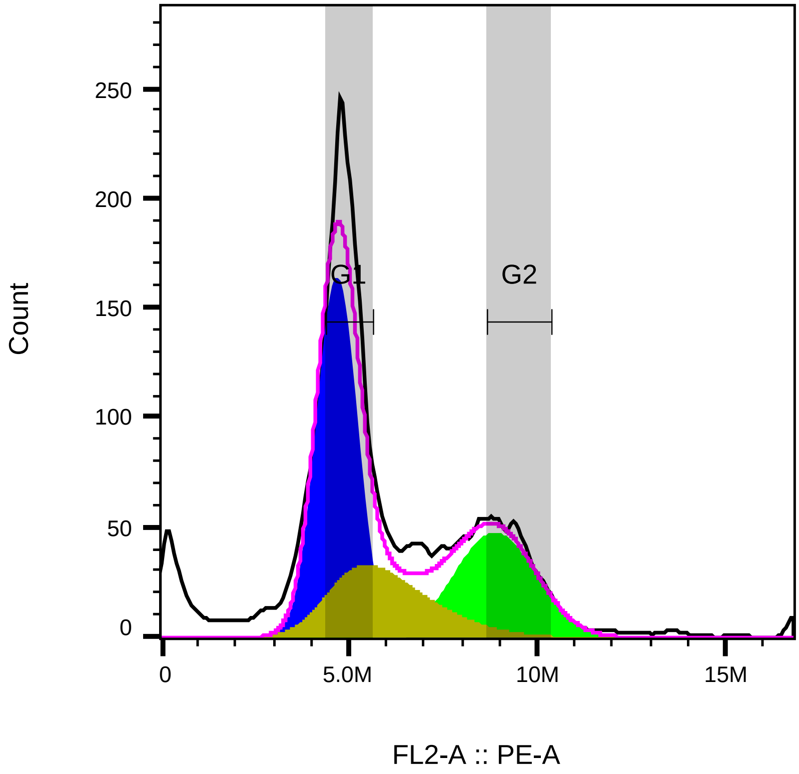

4.fcs
Cell Cycle
9357

RMSD : 3.49
%G1 : 42.6
%S : 20.6
%G2 : 24.9
G1 Mean : 4.70E6
G2 Mean : 8.88E6
G1 CV : 16.0
G2 CV : 17.0
% less G1 : 6.73
% greater G2 : 1.19

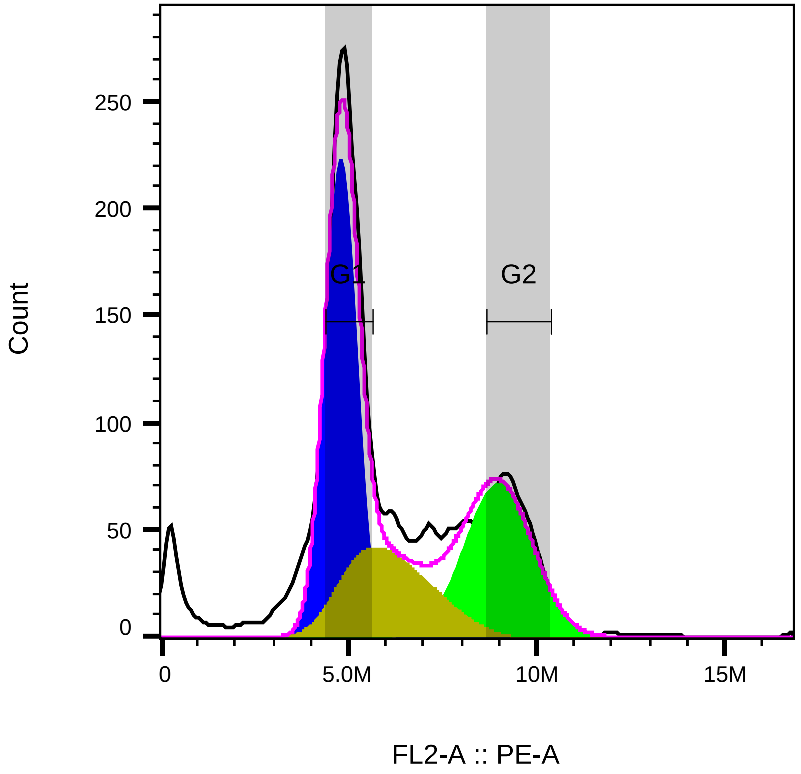

5.fcs
Cell Cycle
9974

RMSD : 3.44
%G1 : 40.5
%S : 20.5
%G2 : 27.8
G1 Mean : 4.82E6
G2 Mean : 8.99E6
G1 CV : 12.3
G2 CV : 14.1
% less G1 : 7.92
% greater G2 : 0.73

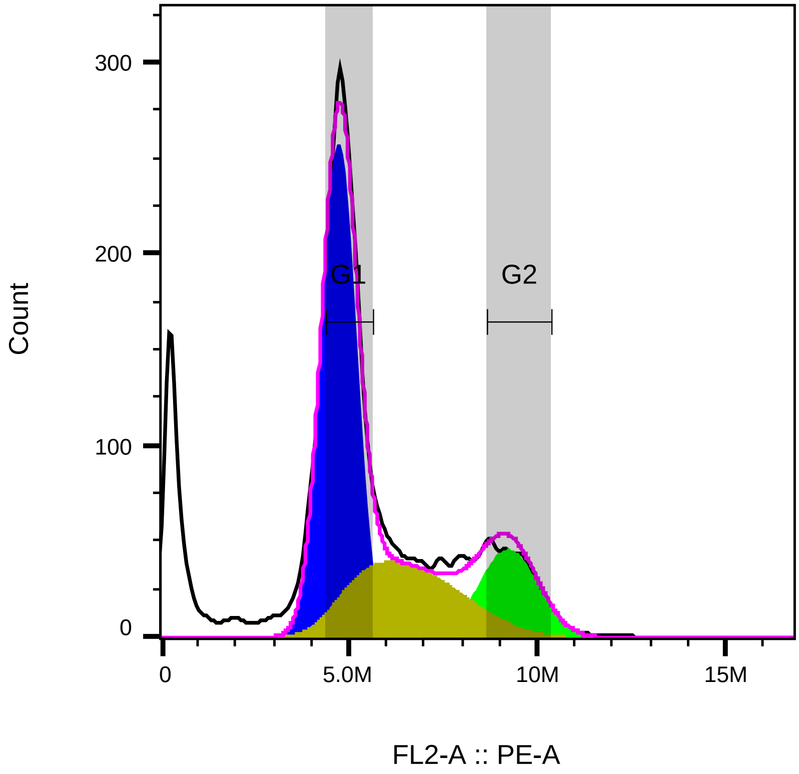

6.fcs
Cell Cycle
10410

RMSD : 6.56
%G1 : 46.4
%S : 22.3
%G2 : 13.8
G1 Mean : 4.75E6
G2 Mean : 9.24E6
G1 CV : 13.7
G2 CV : 11.8
% less G1 : 15.0
% greater G2 : -0.14

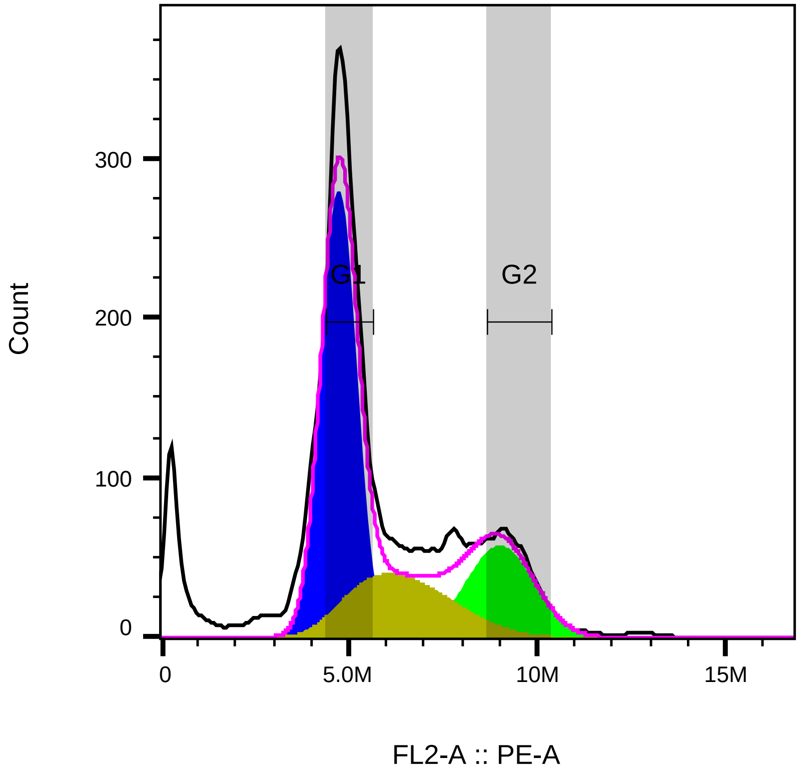

7.fcs
Cell Cycle
12761

RMSD : 5.50
%G1 : 48.0
%S : 19.8
%G2 : 18.8
G1 Mean : 4.75E6
G2 Mean : 9.01E6
G1 CV : 13.9
G2 CV : 14.0
% less G1 : 10.5
% greater G2 : 0.45

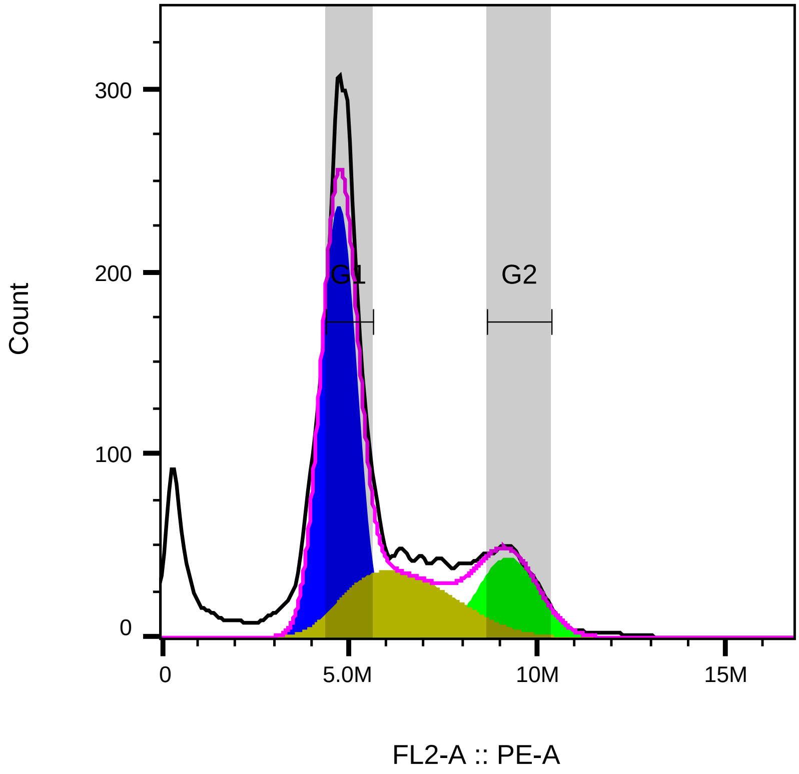

8.fcs
Cell Cycle
10588

RMSD : 4.76
%G1 : 48.2
%S : 21.9
%G2 : 14.9
G1 Mean : 4.75E6
G2 Mean : 9.22E6
G1 CV : 14.0
G2 CV : 12.1
% less G1 : 12.0
% greater G2 : 0.43

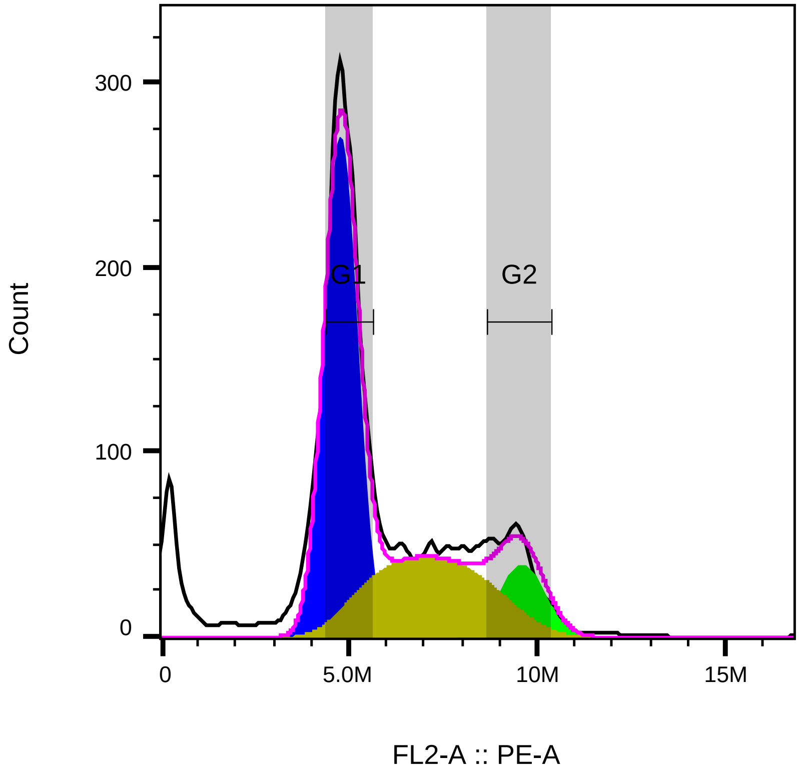

9.fcs
Cell Cycle
10423

RMSD : 4.16
%G1 : 48.7
%S : 28.8
%G2 : 9.62
G1 Mean : 4.80E6
G2 Mean : 9.60E6
G1 CV : 13.1
G2 CV : 8.86
% less G1 : 10.4
% greater G2 : -0.19

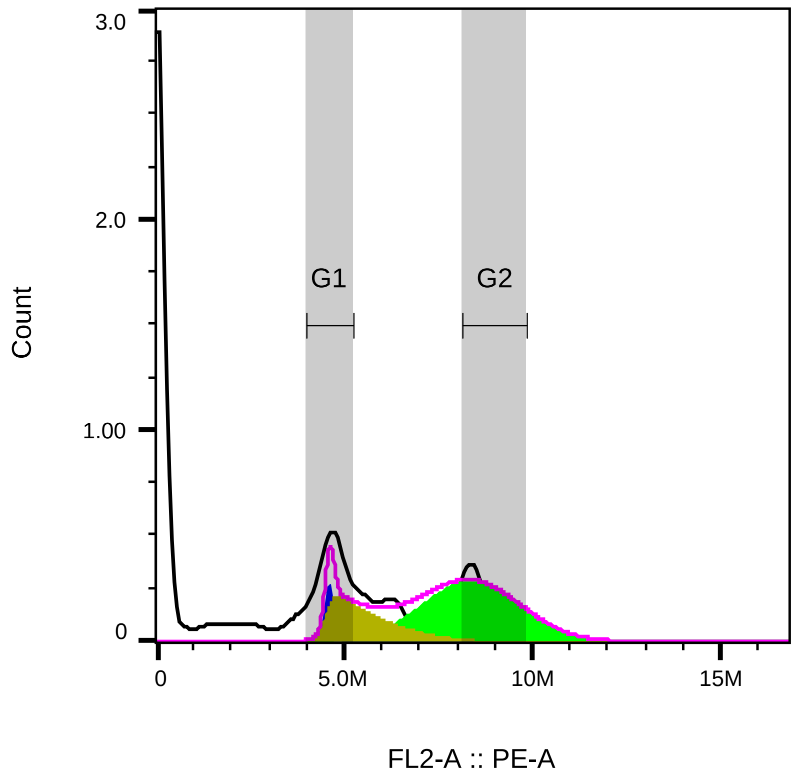

10.fcs
Cell Cycle
42.0

RMSD : 0.18
%G1 : 7.62
%S : 35.7
%G2 : 90.8
G1 Mean : 4.63E6
G2 Mean : 8.38E6
G1 CV : 3.53
G2 CV : 22.0
% less G1 : 52.2
% greater G2 : -36.7

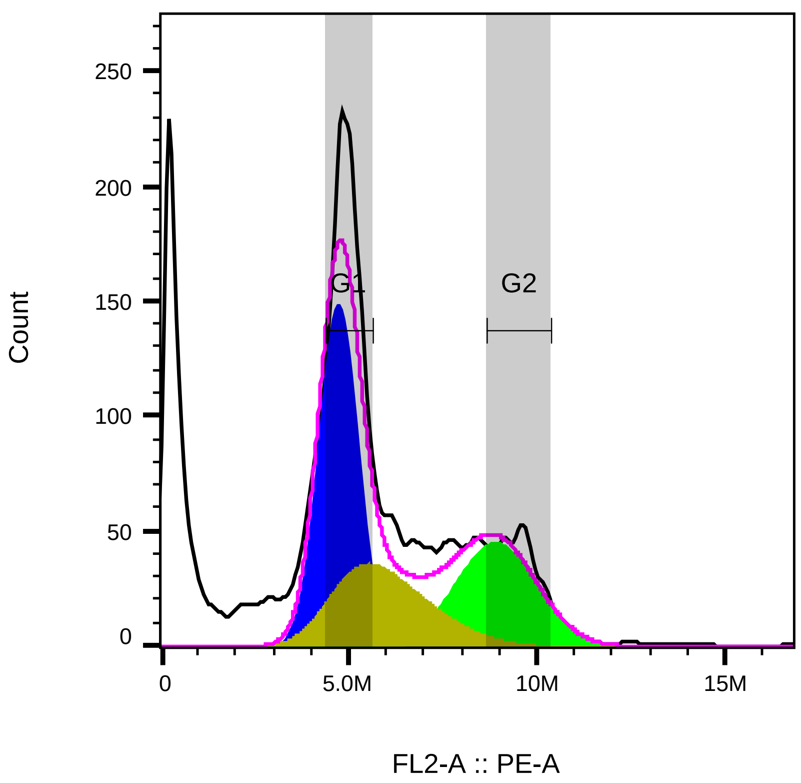

11.fcs
Cell Cycle
10764

RMSD : 9.40
%G1 : 34.1
%S : 19.5
%G2 : 21.3
G1 Mean : 4.76E6
G2 Mean : 8.91E6
G1 CV : 15.5
G2 CV : 17.1
% less G1 : 21.5
% greater G2 : 0.20

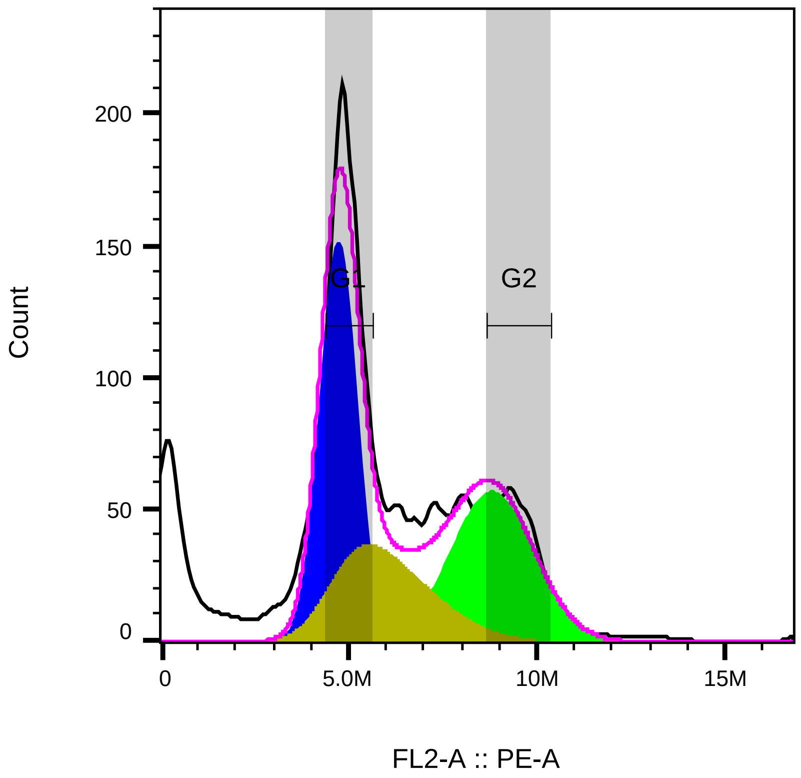

12.fcs
Cell Cycle
9178

RMSD : 4.50
%G1 : 34.7
%S : 20.7
%G2 : 29.0
G1 Mean : 4.75E6
G2 Mean : 8.82E6
G1 CV : 14.6
G2 CV : 17.6
% less G1 : 11.8
% greater G2 : 0.18

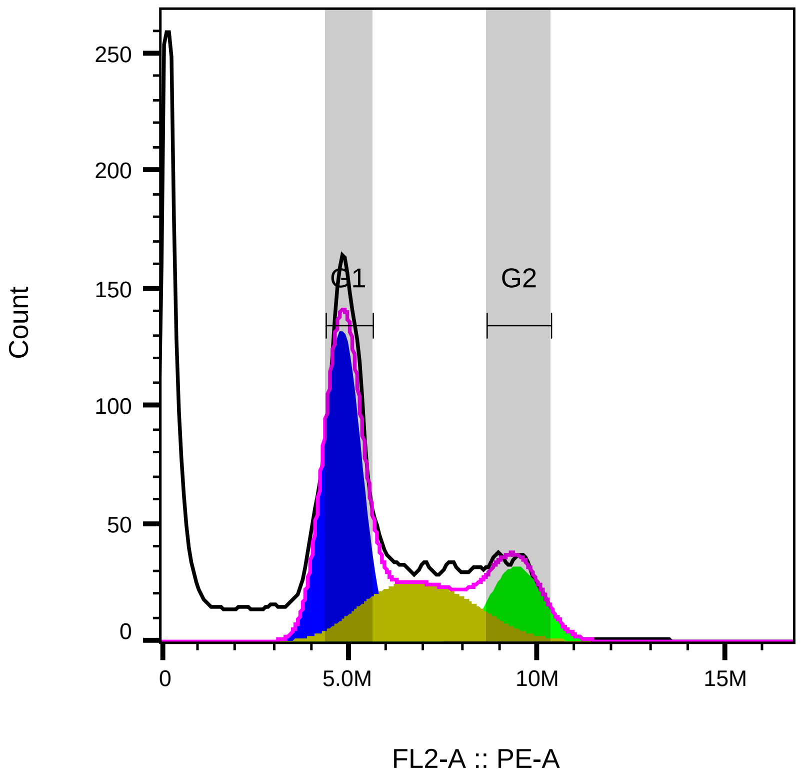

13.fcs
Cell Cycle
8558

RMSD : 11.6
%G1 : 34.7
%S : 19.1
%G2 : 11.6
G1 Mean : 4.84E6
G2 Mean : 9.46E6
G1 CV : 14.5
G2 CV : 10.3
% less G1 : 32.8
% greater G2 : 0.16

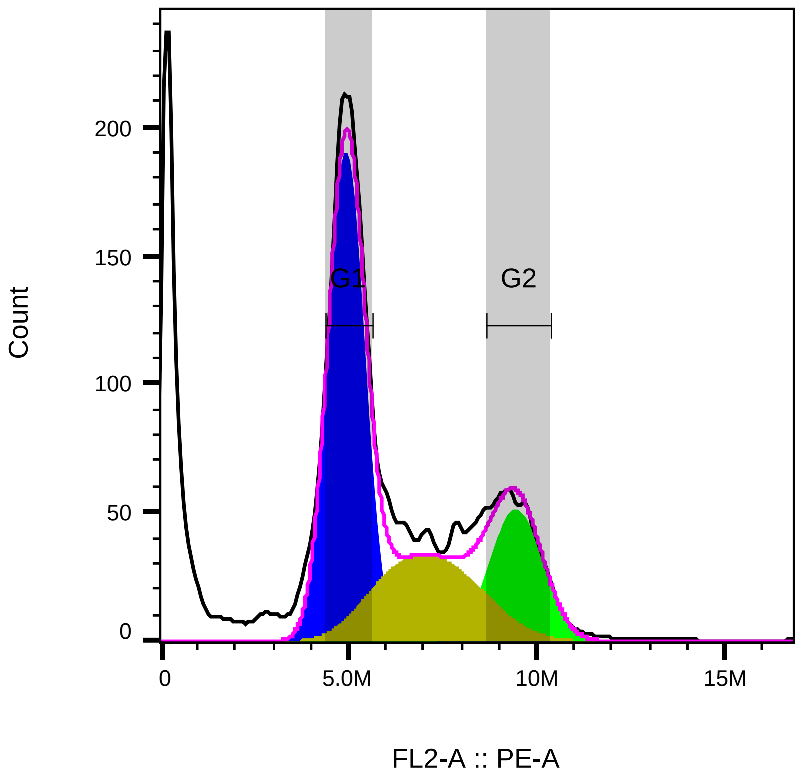

14.fcs
Cell Cycle
10079

RMSD : 9.81
%G1 : 40.2
%S : 20.1
%G2 : 15.3
G1 Mean : 4.95E6
G2 Mean : 9.45E6
G1 CV : 13.8
G2 CV : 10.3
% less G1 : 22.9
% greater G2 : 0.038

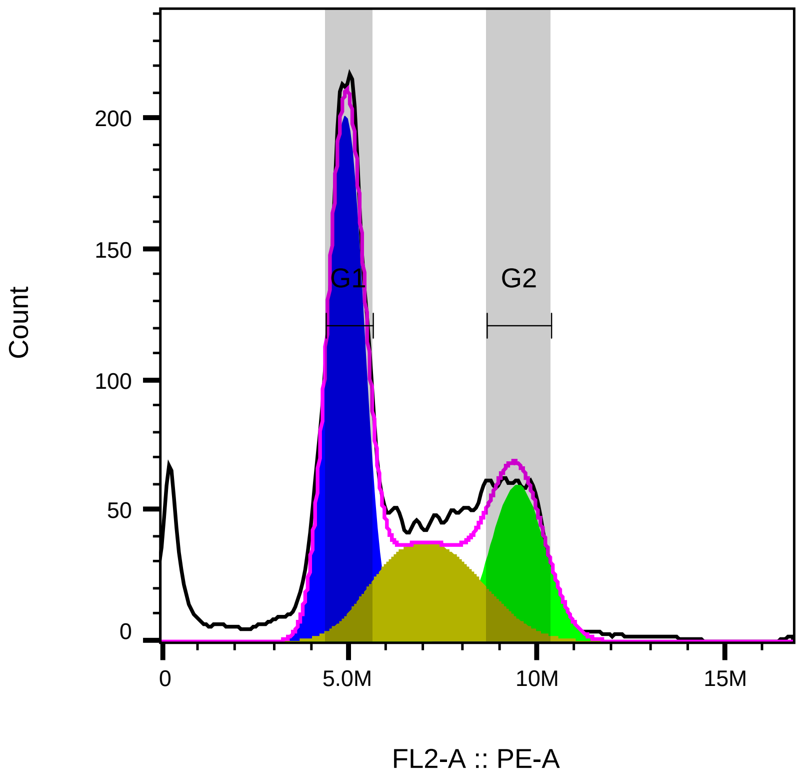

15.fcs
Cell Cycle
9235

RMSD : 3.30
%G1 : 44.6
%S : 24.0
%G2 : 19.8
G1 Mean : 4.93E6
G2 Mean : 9.49E6
G1 CV : 13.7
G2 CV : 10.7
% less G1 : 9.29
% greater G2 : 0.42

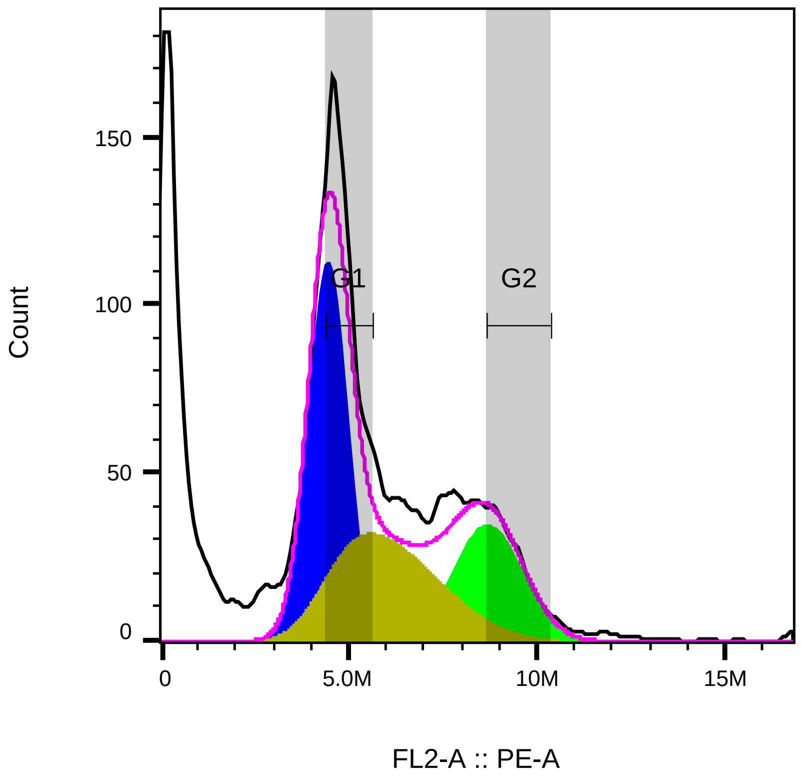

16.fcs
Cell Cycle
8779

RMSD : 8.71
%G1 : 30.9
%S : 23.6
%G2 : 16.8
G1 Mean : 4.48E6
G2 Mean : 8.70E6
G1 CV : 16.4
G2 CV : 14.9
% less G1 : 24.7
% greater G2 : 0.41
